# Genetic variation and association mapping for 12 agronomic traits in *indica* rice

**DOI:** 10.1186/s12864-015-2245-2

**Published:** 2015-12-16

**Authors:** Qing Lu, Mengchen Zhang, Xiaojun Niu, Shan Wang, Qun Xu, Yue Feng, Caihong Wang, Hongzhong Deng, Xiaoping Yuan, Hanyong Yu, Yiping Wang, Xinghua Wei

**Affiliations:** State Key Laboratory of Rice Biology, China National Rice Research Institute, Hangzhou, 310006 China; College of Agricultural Sciences, Shanxi Agricultural University, Taigu, 030801 China

**Keywords:** Genome-wide association study, Morphological trait, Plant architecture, Rice (*Oryza sativa* L.)

## Abstract

**Background:**

Increasing rice (*Oryza sativa* L.) yield is a crucial challenge for modern agriculture. The ideal plant architecture is considered to be critical to enhance rice yield. Elite plant morphological traits should include compact plant type, short stature, few unproductive tillers, thick and sturdy stems and erect leaves. To reveal the genetic variations of important morphological traits, 523 germplasm accessions were genotyped using the Illumina custom-designed array containing 5,291 single nucleotide polymorphisms (SNPs) and phenotyped in two independent environments. Genome-wide association studies were performed to uncover the genotypic and phenotypic variations using a mixed linear model.

**Results:**

In total, 126 and 172 significant loci were identified and these loci explained an average of 34.45 % and 39.09 % of the phenotypic variance in two environments, respectively, and 16 of 298 (~5.37 %) loci were detected across the two environments. For the 16 loci, 423 candidate genes were predicted in a 200-kb region (±100 kb of the peak SNP). Expression-level analyses identified four candidate genes as the most promising regulators of tiller angle. Known (*NAL1* and *Rc*) and new significant loci showed pleiotropy and gene linkage. In addition, a long genome region covering ~1.6 Mb on chromosome 11 was identified, which may be critical for rice leaf architecture because of a high association with flag leaf length and the ratio of flag leaf length and width. The pyramid effect of the elite alleles indicated that these significant loci could be beneficial for rice plant architecture improvements in the future. Finally, 37 elite varieties were chosen as breeding donors for further rice plant architectural modifications.

**Conclusions:**

This study detected multiple novel loci and candidate genes related to rice morphological traits, and the work demonstrated that genome-wide association studies are powerful strategies for uncovering the genetic variations of complex traits and identifying candidate genes in rice, even though the linkage disequilibrium decayed slowly in self-pollinating species. Future research will focus on the biological validation of the candidate genes, and elite varieties will also be of interest in genome selection and breeding by design.

**Electronic supplementary material:**

The online version of this article (doi:10.1186/s12864-015-2245-2) contains supplementary material, which is available to authorized users.

## Background

Plant morphological traits have important effects on rice yield, and elite plant architecture should include short stature, few unproductive tillers, thick and sturdy stems, more grains per panicle and erect leaves [1]. Moreover, these traits are controlled by multiple quantitative loci. Until now, most quantitative trait loci (QTLs) were identified using bi-parental linkage mapping populations. For tiller number and angle, *MOC1*, a gene that is important in the control of rice tillering, was mapped on chromosome 6 [2]. *TAC1*, a major tiller angle (TA) gene, was identified on chromosome 9 [3]. *LA1*, which regulates shoot gravitropism to control TA, was detected on chromosome 11 [4]. *PROG1*, which controls TA and the number of tillers, was mapped on chromosome 7 [5, 6]. For plant height, the green revolution gene, *sd1*, was mapped on chromosome 1 and encodes GA20_ox_ [7]. Moreover, *IPA1*, which profoundly changes rice plant architecture and substantially enhances rice grain yield, was located on chromosome 8 [8]. Additionally, *D53* acts as a repressor of strigolactone signaling, and a d53 mutant produced an exaggerated number of tillers to control rice plant architecture [9, 10]. For leaf architecture, a classic gene, *NAL1* with pleiotropic effects on multiple traits, including polar auxin transport and vascular patterns, was characterized on chromosome 4 [11–14]. A great many of the flag leaf architecture QTLs (or genes) have been isolated, such as *LC1* [15], *qFLL6.2* [16], *qFL1* [17], *qFLW7.2* [18] and *qLSCHL4* [19]. For panicle type, *qPE9-1* [20] and *EP2* [21] were two erect panicle QTLs, and *sp1* leads to a short-panicle phenotype [22].

Although linkage mapping is a powerful method to detect QTLs, the discovery of QTLs is limited by the material used. Based on the historic recombination in diverse germplasm indicated by the presence of linkage disequilibrium (LD) between markers and QTLs across the genome, genome-wide association studies (GWASs) are considered to be an excellent way to uncover the genetic variations of complex traits using a large natural population [23]. In the past few years, GWASs were successfully applied to the dissection of complex traits in humans [24] and animals [25]. In 2010, GWASs were successfully applied to the analysis of 107 phenotypes in *Arabidopsis thaliana* inbred lines [26]. Subsequently, GWAS research has been implemented in crops such as rice [27–32], maize [33, 34], soybean [35, 36] and wheat [37].

Compared with a QTL linkage analysis, GWAS is commonly performed using a large unrelated diverse germplasm to increase the diversity of alleles and haplotypes [38]. A high-density SNP map and comprehensive HapMap have been constructed in rice (*Oryza sativa* L.) [27]. However, limited studies have been carried out to analyze rice morphological traits systematically based on the high-density map. In our study, we used a systematic GWAS in 523 diverse accessions for 12 phenotypes in two environments, Lingshui (LS; N 18°32′, E 110°01′), Hainan Province and Hangzhou (HZ; N 30°15′, E 120°12′), Zhejiang Province, China in 2014. The goals of this study were (1) to uncover the genetic architecture of morphological traits, including tiller number, TA, flag leaf architecture and panicle characteristics; (2) to identify a substantial number of significant loci related to morphological traits for candidate gene identification and elite allele analysis; and (3) to discover elite breeding lines for rice plant architecture and yield improvement. The results will aid our understanding of mechanisms underlying rice morphological traits, and provide valuable theoretical knowledge for rice genomic selection and breeding by design.

## Methods

### Plant materials

**Fig. 1:**
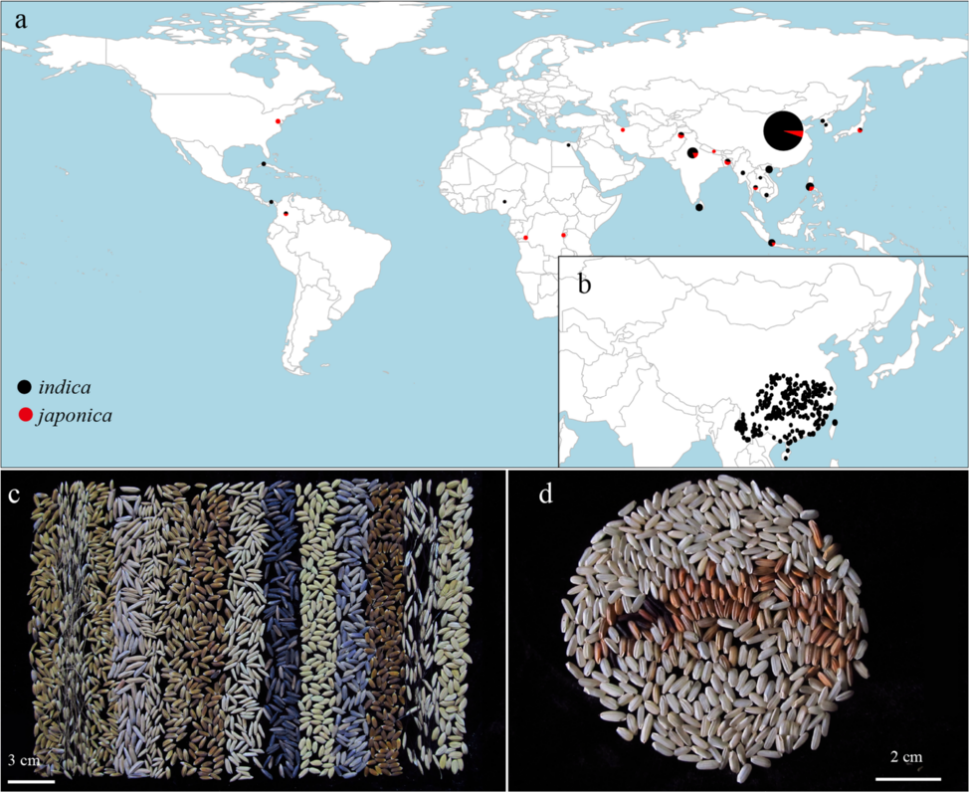
Material distribution and germplasm diversity. **a** Geographic origins of 523 rice samples. The pie charts on the world map represent the country-specific distribution. The size of the pie chart shows the sample size, and colors within each pie chart reflect the proportion of *indica* (black) and *japonica* (red); **b** Most of the *indica* accessions are distributed in southern China; **c** Grain with rich phenotypic variations; **d** Brown rice with different pericarp colors

The GWAS experiment comprised 523 rice germplasm accessions (469 *indica* and 54 *japonica*), which were collected from 25 countries, representing the major rice-growing regions of the world (Fig. 1a; Additional file 1: Table S1). To limit the influence of strong population differentiation between the two subspecies, we only chose 469 *indica* accessions, most of which were from southern China (Fig. 1b). These accessions, with three replications, were planted in a randomized complete block designed of 6 column × 6 row, with spacing of 20 cm among the plants in LS and HZ in 2014. For each block, the four plants in the middle block were selected to evaluate phenotypes to eliminate the marginal effects. The measurement methods of 12 morphological traits are summarized in Additional file 2: Figure S1.

### SNP genotype calling

Rice genomic DNA was extracted from the young leaf tissue of each of the 523 accessions, which had been grown in the experimental field of the China National Rice Research Institute in Hangzhou in 2014. All of the accessions were genotyped using Illumina custom-designed arrays by following the Infinium HD Assay Ultra Protocol [http://support.web.illumina.com/downloads/infinium_hd_ultra_assay_protocol_guide_(11328087_b).html] (Illumina, Inc., San Diego, CA). The array consisted of 5,291 single nucleotide polymorphisms (SNPs) that were chosen from the Rice Haplotype Map Project Database (http://202.127.18.221/RiceHap2/index.php) [27]. Genotypes of these accessions were called using Genome Studio (Illumina, USA). The quality of each SNP was confirmed manually, and a few of the SNPs of low quality and a minor allele frequency < 5 % were removed from the dataset. Finally, 4,136 SNPs were used for the GWAS analysis (Additional file 3: Table S2).

### Genotypic data analysis

Major allele frequency, gene diversity, polymorphic information content (PIC) (Additional file 3: Table S2) and population differentiation (*F*_ST_) among different subpopulations (Additional file 4: Table S3) were calculated using PowerMarker version 3.25 [39]. Based on the Bayesian Markov Chain Monte Carlo (MCMC) Program, the genetic component of each accession and the number of subpopulations (K), ranging from 1 to 12 in all of the population (and 1 to 15 in the *indica* subpopulation), were inferred using STRUCTURE version 2.2 [40]. In the analysis, five independent runs with a burn-in period of 10,000 steps and 100,000 MCMC replications were implemented. The ancestry model allowed for population mixture and correlated allele frequencies. Both the value of log likelihood [LnP(D)] and an *ad hoc* statistic ΔK [41] were used to reveal the suitable population structure. The results of the five independent runs were integrated using the CLUMPP software [42] and then generated as a Q-matrix for further association mapping. A neighbor-joining (NJ) tree was constructed based on Nei’s genetic distance [43] between pairwise individuals under PowerMarker version 3.25 [39]. Furthermore, according to the Nei’s genetic distance matrix, a principal component analysis (PCA) was performed using NTSYSpc version 2.1 [44]. Finally, the pairwise relatedness coefficients were calculated using SPADiGe version 1.4c with the negative values among individuals set as zero and then all the values were doubled to obtain the K matrix for further analysis [45].

### Estimation of LD decay

The LD parameter *r*^*2*^ was computed using TASSEL version 4.0 [46] to evaluate the level of LD between linked SNPs. The LD decay rate was measured as the chromosomal distance at which the average pairwise correlation coefficient dropped to half of its maximum value [27, 35]. The LD block was calculated using Haploview 4.2 [47].

### Phenotypic variation and correlation

A total of 12 morphological traits were evaluated in two environments. The statistical analyses of mean, standard error (SE) and broad-sense heritability (*H*_*B*_^2^) were calculated using Microsoft Office Excel 2007. The broad-sense heritability was computed through an analysis of variance using the following formula:

$$ {H}_B^2={\sigma}_g^2/\left({\sigma}_g^2+{\sigma}_e^2\right), $$

where *σ*_*g*_^2^ is genetic variance and *σ*_*e*_^2^ is error variance. Correlation coefficients among the 12 agronomic traits were computed by R (http://www.r-project.org/). An ANOVA was carried out to evaluate the effects of genotype (G), environment (E) and genotype × environment (G × E) using SAS system 9.0 (SAS, Inc., Cary, NC, USA), and the percentage of phenotypic variation explained by population structure was also carried out in SAS 9.0.

### Genome-wide association

An association analysis was performed using TASSEL version 4.0 [46], and the EMMA [48] and P3D [49] algorithms were performed to reduce computing time. Four models including GLM, Q, K, and Q + K were used to evaluate the effects of population structure and relative kinship. The GLM model: without any factor as covariate; the Q model: Q-matrix as covariate; the K model: K matrix as covariate; and the Q + K model: both Q and K matrix as covariates. The GLM and Q models were performed using a general linear model program. However, the K and Q + K models were computed under a mixed linear model program [50, 51]. The two model program equations can be expressed as

$$ y=X\alpha +e\kern0.5em  and\kern0.5em y=X\alpha +Q\beta +K\mu +e, $$

respectively, where *y* was the vector of phenotypic observation, α was the vector of SNP effects, β was the vector of population effects, μ was the vector of kinship effects, *e* was the vector of residual effects, *Q* was the matrix from STRUCTURE relating *y* to β, and *X* and *K* were incidence matrices of 1 s and 0 s relating *y* to α and μ, respectively [35, 50]. For the comparison, we adopted two significant thresholds, the Minimum Bayes factor (BF = −*e × p × ln*(*p*)) [52, 53] and the Bonferroni (*P* = *a/n*) [54, 55]. In this study, we used 1/n (*a* = 1, 1/4,136 = 2.4 × 10^−4^) as the cutoff for all traits except for pericarp color (*a* = 0.001, 0.001/4,136 = 2.4 × 10^−7^).

For the significant marker-trait associations, the elite and null alleles were used to determine the choice of elite accessions for further breeding by design. The formula for calculating phenotypic effect of alleles can be described as

$$ a=\left({\displaystyle {\sum}_{i=1}^n{\chi}_i}\right)/n-\left({\displaystyle {\sum}_{j=1}^m{y}_j}\right)/m, $$

where *x*_*i*_ was the phenotypic value of accession carrying the *i* allele, *y*_*j*_ was the phenotypic value accession carrying the *j* allele, and *n* and *m* represented the number of accessions. If *a* was positive, then the *i* allele was defined as an elite allele with an increasing effect and the *j* allele was defined as the null allele with a decreasing effect, and vice versa. In our study, we chose parental accessions with elite or null alleles based on the characteristics of different traits.

### Candidate gene expression profiling

The expression levels of 14 TA candidate genes were measured by quantitative real-time PCR (qPCR) using eight accessions with large TAs at the seedling, early and later tillering stages, respectively. Total RNA was extracted from the base of tiller tissue using a MiniBEST Plant RNA Extraction kit (Takara Bio Inc, Japan). Complementary DNA (cDNA) was synthesized by PrimeScript RT Master Mix (Takara Bio Inc, Japan). The reaction was performed on an ABI Step-One Plus Real Time PCR System (Applied Biosystems, USA). The expression level of *actin* was used to standardize the data for each run. The expression experiment was performed at three replications for each sample. The qPCR primers and materials are summarized in Additional file 5: Table S4. Duncan’s multiple comparison was performed by SPSS 13.0 (Chicago, IL) at 0.01 significance level.

## Results

### Material distribution and genotyping array

**Fig. 2:**
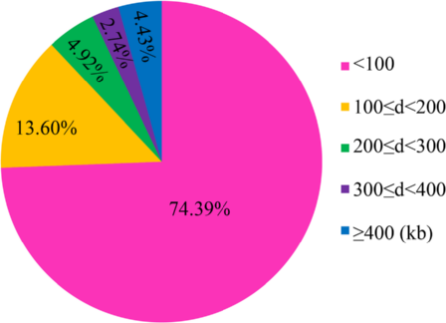
Proportion of the 4,136 SNPs categorized by inter-SNP (the adjacent SNPs) distances. d represents the distance between two adjacent SNPs

Rice germplasm resources maintain most of their genetic diversity during the process of domestication (Fig. 1c and d). In our study, 523 accessions of *O. sativa* collected from 25 countries (Fig. 1a; Additional file 1: Table S1) were genotyped using the Illumina custom-designed genotyping array containing 5,291 SNPs. The SNPs with minor allele frequencies < 5 % were deleted from the dataset to avoid problems of spurious LD and false positive associations. Finally, 4,136 SNPs of high genotyping quality were used for the GWAS analysis (Additional file 3: Table S2). For these SNPs, 74.39 % inter-marker distances were less than 100 kb and only 4.43 % were more than 400 kb (Fig. 2).

### Population divergence

**Fig. 3:**
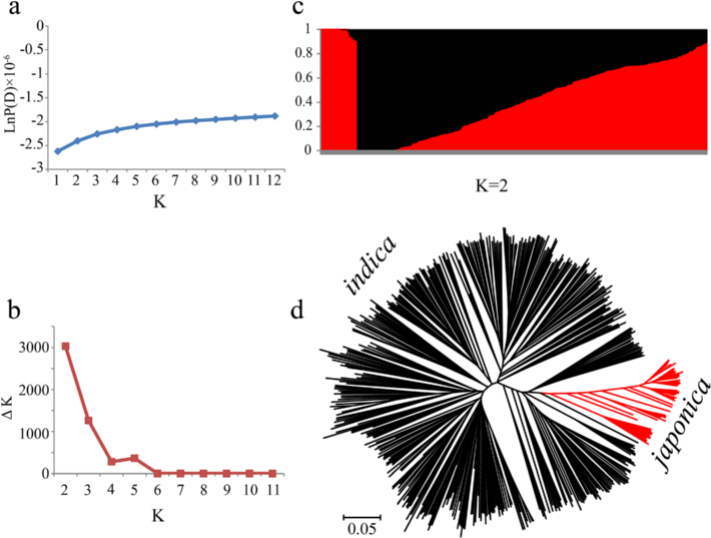
Population structure of 523 rice samples. **a** Mean LnP(D) values plotted as the number of subpopulations; **b** ΔK values plotted as the number of subpopulations; **c** Subpopulations inferred using STRUCTURE; **d** NJ tree based on Nei’s genetic distances

We first calculated the genetic component of each variety using STRUCTURE (K = 1 to 12). The results showed that the LnP(D) value for each given K, from 1 to 12, increased but did not show a maximum value (Fig. 3a). The value of Evanno’s ΔK showed the highest peak at K = 2 (Fig. 3b). Therefore, we speculated that the two subspecies, *indica* and *japonica*, were present in major groups (Fig. 3c), and the result was also confirmed by the NJ tree (Fig. 3d). In addition, the *F*_ST_ between *indica* and *japonica* was 0.56, suggesting a strong level of differentiation between the two subspecies (Additional file 4: Table S3), which was in close agreement with a previous report [27].

**Fig. 4:**
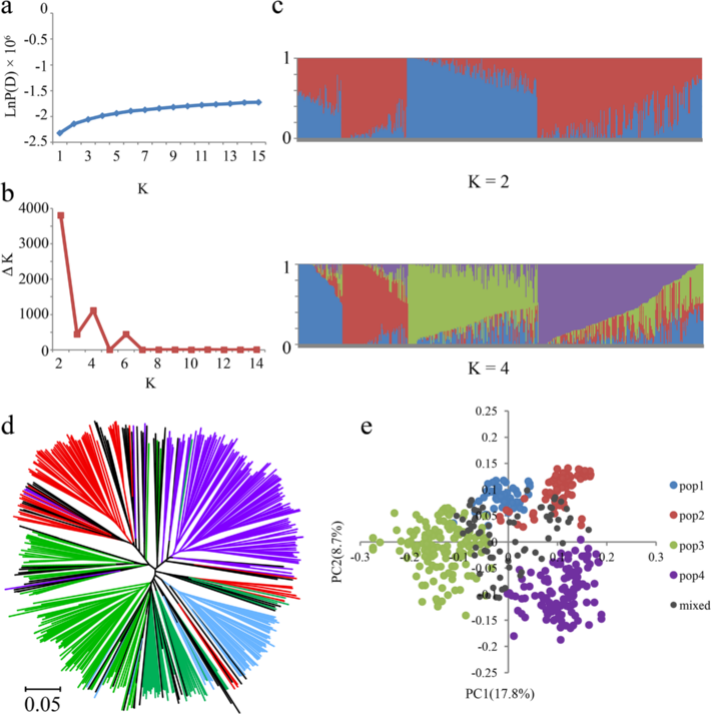
Population structure of 469 *indica* samples. **a** Mean LnP(D) values plotted as the number of subpopulations; **b** ΔK values plotted as the number of subpopulations; **c** Subpopulations (K = 2 and K = 4) inferred using STRUCTURE; **d** NJ tree based on Nei’s genetic distances. Blue, red, green, purple and black represent POP1, POP2, POP3, POP4 and Mixed, respectively; **e** Principal component analysis

Given the strong population differentiation between *indica* and *japonica* subspecies, only 469 *indica* accessions, most of which were distributed in southern China (Fig. 1b), were chosen for further analyses. Similarly, we first ran STRUCTURE (K = 1 to 15) on the panel to evaluate the number of subpopulations, and the genetic component of each variety is shown in Additional file 6: Table S5. The LnP(D) value increased continuously with K values of 1 to 15. Meanwhile, the top two peaks of Evanno’s ΔK appeared at K = 2 and K = 4 (Fig. 4a and b). This result suggested that the genetic structure of the 469 *indica* accessions had two or four classifications, which was supported by the occurrence of two subpopulations (K = 2) and four groups under two subpopulations (K = 4; Fig. 4c). When K = 2, the geographic distribution analyses showed that the Group 1 accessions were distributed in the Yangtze River basin, and the Group 2 accessions were distributed in both the Pearl River basin and southeastern Asia (Additional file 7: Figure S2a). When K = 4, the first subpopulation, POP1, was distributed in the Yunnan, Guizhou and Sichuan Provinces. POP2 was distributed in the southeastern coastal areas of China. POP3 was distributed in the Middle-Lower Yangtze Plains and POP4 was distributed in southeastern Asia (Additional file 7: Figure S2b). Furthermore, we constructed a NJ tree based on Nei’s genetic distances (Fig. 4d). There were four clear clades (without considering mixed subpopulation) that represented the four subpopulations (blue, red, green, and purple). Finally, the PCA demonstrated that the top two eigenvectors clearly separated these accessions into four subpopulations (without considering mixed subpopulation) shown as POP1, POP2, POP3, and POP4 (Fig. 4e). PC1 and PC2 accounted for 17.8 % and 8.7 % of the genetic variation, respectively. Combined with the results of the NJ tree and PCA, we concluded that four suitable subpopulations (POP1 to POP4) can be obtained in the 469 *indica* accessions. Moreover, the *F*_ST_ values of the four subpopulations ranged from 0.05 to 0.25, indicating a moderate level of subpopulation differentiation (Additional file 4: Table S3).

### Relative kinship between *indica* individuals

**Fig. 5:**
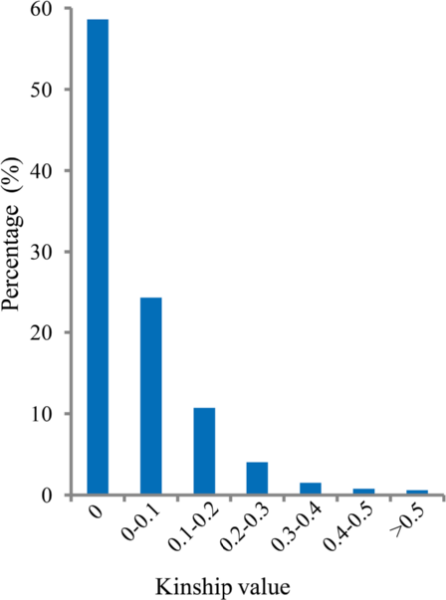
Distribution of pairwise relative kinship coefficients

Most of the pairwise relative kinship values were less than 0.1 (Additional file 8: Figure S3). A total of 58.6 % of the pairwise kinship coefficients were zero, and about 24.4 % of the kinship values ranged from 0 (excluding 0) to 0.1. A fraction of kinship values (16.7 %) showed various degrees of relatedness from 0.1 (excluding 0.1) to 0.5, and only 0.3 % of the values were larger than 0.5 (Fig. 5). This pattern of relative kinship demonstrated that there was no or weak relatedness in the complex *indica* panel.

### LD analysis

**Fig. 6:**
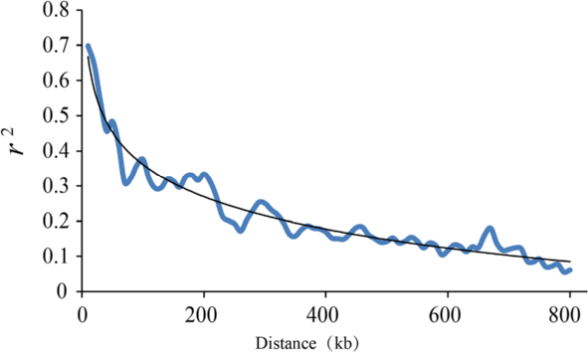
Estimation of genome-wide average LD decay distance in the *indica* panel

The pairwise SNP LD parameter *r*^2^ was analyzed using TASSEL 4.0 [46]. A regression analysis between *r*^2^ and genetic distance indicated that they fitted the equation *y* = −0.133*ln*(*x*) + 0.9733 (*R*^*2*^ = 0.9422) (Fig. 6). Therefore, the genome-wide LD decay distance was ~109.37 kb at which the *r*^*2*^ dropped to half its maximum value (*r2 max*) (Additional file 9: Table S6). Then, according to the same strategy, the LD decay rate of each of the 12 chromosome was estimated. The shortest LD decay distance was 96.15 kb on chromosome 5, and the longest was 421.39 kb on chromosome 7. The average decay distance was 214.69 kb (Additional file 9: Table S6). These results were consistent with previous studies that cultivated rice had a long-range LD decay distance from ~100 kb to ~200 kb [27, 56]. Given that most of the inter-marker distances were less than 100 kb (Fig. 2), we expected to have a reasonable power to detect common large-effect variations in the *indica* panel.

### Phenotypic variation and correlation

A wide range of phenotypic variations were observed for the 12 morphologic traits in LS and HZ (Table 1; Additional file 10: Table S7). The average *H*_*B*_^*2*^ was 71.43 %, ranging from 36.49 % for flag leaf length (FLL) to 99.61 % for pericarp color (PC). The average percentage of phenotypic variation explained by population structure was 21.76 %, indicating that phenotypic variation was influenced by population structure. Hence, the Q-matrix was necessary as a covariate for the GWAS analysis to control false positive associations.

**Table 1:** Phenotypic variation, explanation and broad-sense heritability of each trait in the *indica* panel

Trait ^a^	Environment ^b^	Mean ± S.E ^c^	Range	*R*_*Q*_^*2*^ (%) ^d^	*H*_*B*_^*2*^ (%) ^e^
TN	LS	12.47 ± 0.16	4.67 ~ 26.50	30.78	80.08
	HZ	13.29 ± 0.18	5.75 ~ 32.00	24.49	
TA	LS	18.24 ± 0.28	8.00 ~ 35.00	11.06	74.25
	HZ	16.40 ± 0.19	8.67 ~ 31.40	11.34	
PH	LS	110.91 ± 1.20	54.50 ~ 199.50	52.93	77.36
	HZ	134.47 ± 1.45	74.60 ~ 212.60	57.53	
FLL	LS	27.23 ± 0.22	14.00 ~ 40.60	24.77	36.49
	HZ	39.13 ± 0.34	21.88 ~ 68.25	23.21	
FLW	LS	1.45 ± 0.01	0.90 ~ 2.14	19.19	75.66
	HZ	1.61 ± 0.01	1.03 ~ 2.43	14.52	
FLLW	LS	19.18 ± 0.19	9.42 ~ 35.75	25.20	61.53
	HZ	24.74 ± 0.26	12.32 ~ 43.45	21.07	
FLA	LS	1.89 ± 0.06	1 ~ 7	10.57	62.67
	HZ	2.69 ± 0.08	1 ~ 7	37.03	
PN	LS	9.29 ± 0.12	4.83 ~ 22.33	29.24	79.37
	HZ	9.13 ± 0.12	4.60 ~ 27.20	15.64	
PT	LS	2.21 ± 0.03	1 ~ 5	9.74	72.15
	HZ	2.58 ± 0.04	1 ~ 5	24.26	
PL	LS	22.38 ± 0.11	17.20 ~ 30.10	18.95	49.52
	HZ	25.93 ± 0.11	18.42 ~ 32.83	14.97	
PC	LS	-	0 ~ 1	17.26	99.61
	HZ	-	0 ~ 1	14.69	
HC	LS	-	0 ~ 1	8.16	88.46
	HZ	-	0 ~ 1	5.70	
Average				21.76	71.43

^a^ *TN* tiller number, *TA* tiller angle, *PH* plant height, *FLL* flag leaf length, *FLW* flag leaf width, *FLLW* the ratio of flag leaf length and flag leaf width, *FLA* flag leaf angle, *PN* panicle number, *PT* panicle type, *PL* panicle length, *PC* pericarp color, *HC* hull color; ^b^ *LS* Lingshui, *HZ* Hangzhou; ^c^ *S.E* standard error; ^d^ *R*_*Q*_^2^ (%), Percentage of phenotypic variation explained by population structure estimated by Structure; ^e^ *H*_*B*_^2^ (%), Broad-sense heritability; −, No data

ANOVA results indicated that the effects of G, E and G × E were all highly significant (*P* < 0.001) except for the effect of E on panicle number (*P* < 0.05; Table 2). These results implied that rice morphologic traits were highly affected by E.

**Table 2:** ANOVA for 12 agronomic traits

Trait	TN	TA	PH	FLL	FLL	FLLW	FLA	PN	PT	PL	PC	HC
G	***	***	***	***	***	***	-	***	-	***	-	-
E	***	***	***	***	***	***	-	*	-	***	-	-
G × E	***	***	***	***	***	***	-	***	-	***	-	-

*TN* tiller number, *TA*, tiller angle, *PH* plant height, *FLL* flag leaf length, *FLW* flag leaf width, *FLLW* the ratio of flag leaf length and flag leaf width, *FLA* flag leaf angle, *PN* panicle number, *PT* panicle type, *PL* panicle length, *PC* pericarp color, *HC* hull color; * and *** indicate significance levels at 0.05 and 0.001, respectively. -, No data; *G* genotype, *e* environment; G × E, Interaction of genotype and environment

**Fig. 7:**
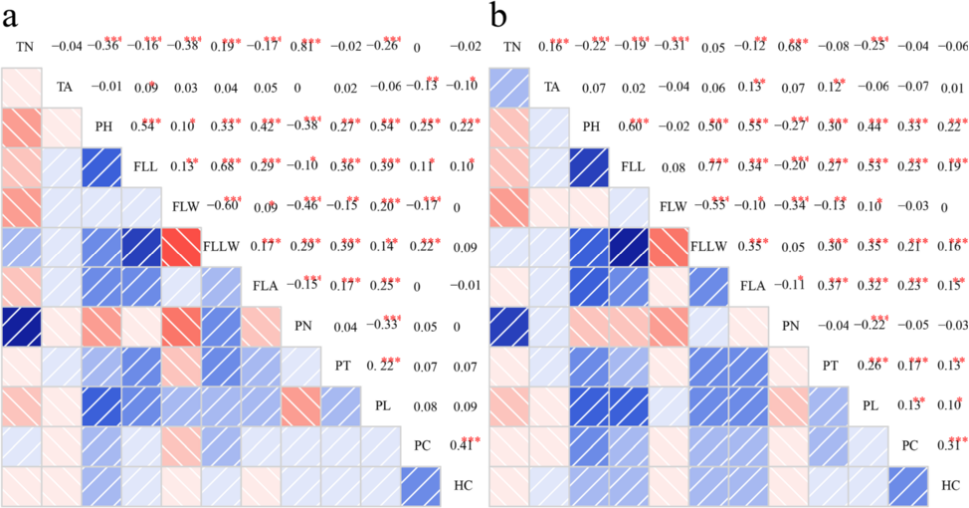
Phenotypic correlation coefficients among 12 agronomic traits in two environments. **a** Phenotypic correlation coefficients in Lingshui; **b** Phenotypic correlation coefficients in Hangzhou. Blue color represents positive correlation, pink color represents negative correlation and the shade of color represents the absolute correlation coefficient value. TN, Tiller number; TA, Tiller angle; PH, Plant height; FLL, Flag leaf length; FLW, Flag leaf width; FLLW, The ratio of flag leaf length and flag leaf width; FLA, Flag leaf angle; PN, Panicle number; PT, Panicle type; PL, Panicle length; PC, Pericarp color; HC, Hull color

Phenotypic correlation coefficients showed that most of the morphological traits were highly significantly (*P* < 0.001 and *P* < 0.01) or significantly (*P* < 0.05) related to each other in LS (Fig. 7a) and HZ (Fig. 7b) in 2014, especially FLL to the ratio of flag leaf length and flag leaf width (FLLW), tiller number (TN) to panicle number (PN), plant height (PH) to FLL, and flag leaf width (FLW) to FLLW. These results demonstrated that rice morphological traits were highly related to each other, and this provides valuable knowledge for rice plant architectural modifications.

### Different model comparison

**Fig. 8:**
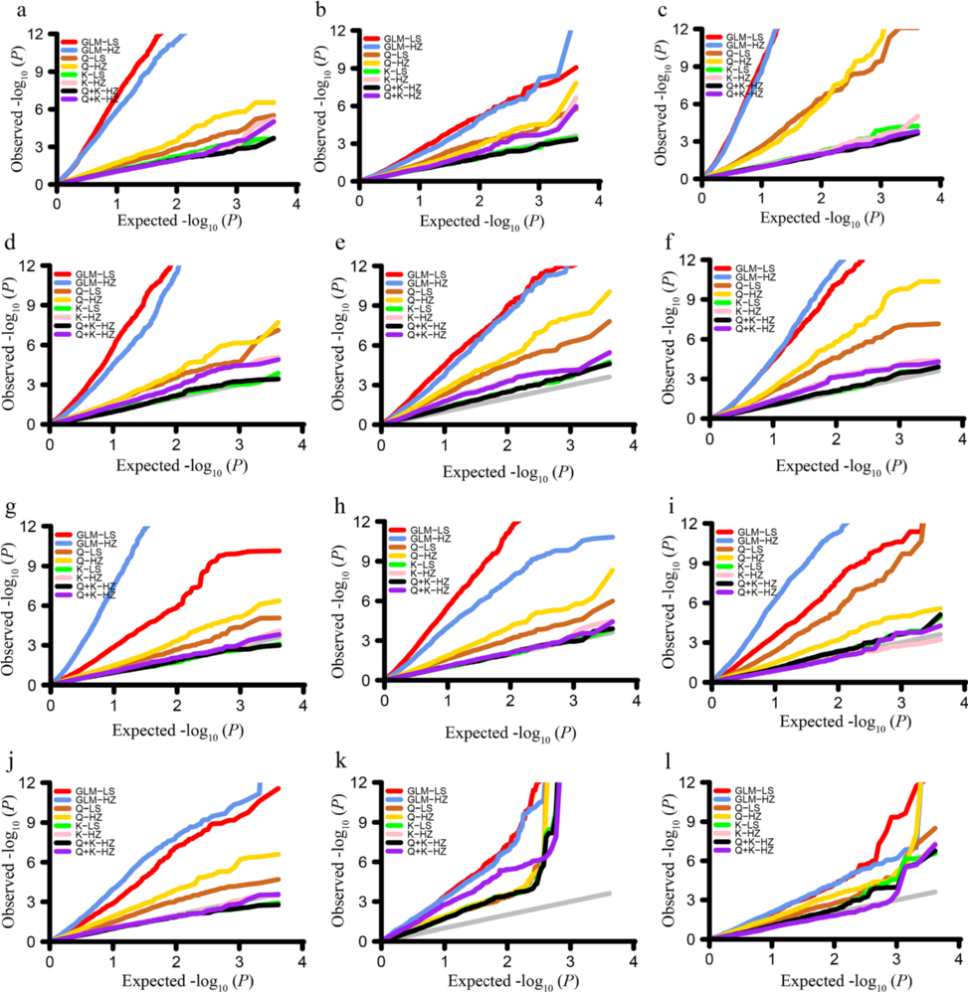
Quantile-quantile plots for 12 morphological traits in two environments. **a** Tiller number; **b** Tiller angle; **c** Plant height; **d** Flag leaf length; **e** Flag leaf width; **f** The ratio of flag leaf length and width; **g** Flag leaf angle; **h** Panicle number; **i** Panicle type; **j** Panicle length; **k** Pericarp color; **l** Hull color. Distribution of *P*-values assuming associations (expected *P*-values) are represented as gray lines; distribution of *P*-values calculated based on the four models (observed *P*-values) are represented as different colored lines; LS, Lingshui; HZ, Hangzhou

**Fig. 9:**
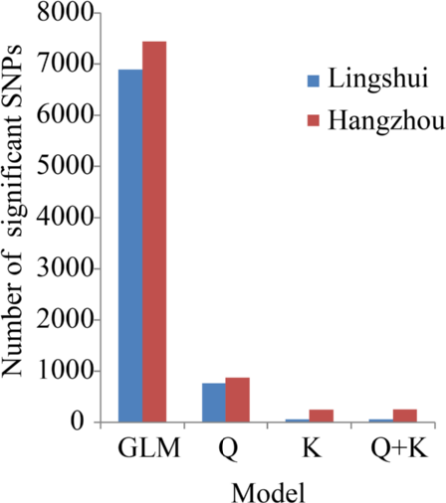
Distribution of the significant SNPs (*P* < 2.4 × 10^−4^) in Lingshui and Hangzhou under the four different association models

To control false positive trait-marker associations, four models, GLM, Q, K and Q + K, were compared with each other using the quantile-quantile (Q-Q) plot shown in Fig. 8. For all 12 traits, the Q, K and Q + K models were significantly better than the GLM model, which did not consider the influence of population structure or relative kinship. Moreover, the K model was better than the Q model. Additionally, the Q + K model, controlling both population structure and relative kinship, was a little better than the K model in reducing the type I errors. For the four models, the number of significant SNPs identified at *P* < 2.4 × 10^−4^ (Bonferroni threshold *a* = 1) is shown in Fig. 9. In LS, the number of significant SNPs was sharply reduced from 6,894 (GLM) to 762 (Q), then to 57 (K) and finally to 56 (Q + K). In HZ, the trend was the same as in LS. These results illustrated that the false positive associations were greatly controlled only when using the Q + K model. Thus, we chose this model for the GWAS analysis.

### Genome-wide association mapping and expression analysis

A total of 456 significant SNPs were identified in LS and HZ. To select major loci among all the significant SNPs, the SNPs with the lowest *P*-values were maintained within a 200-kb window according to the average LD decay rate in the panel. Finally, in LS and HZ, 126 and 172 significant SNPs, including known and new candidate loci, respectively, were identified for the 12 traits and all of the significant loci explained ~34.45 % (10.29 to 72.19 %) and 39.09 % (20.10 to 71.76 %) of the phenotypic variation on average, respectively (Table 3; Additional file 11: Table S8; Additional file 12: Figure S4).

**Table 3:** Summary of associations for 12 traits in the two environments, Lingshui and Hangzhou

	Lingshui			Hangzhou		
Trait ^a^	Num. of sig. locus	*R* ^*2*^ (%) ^b^	Known locus ^c^	Num. of sig. locus	*R* ^*2*^ (%) ^b^	Known locus ^c^
TN	4	16.27		8	35.10	
TA	7	24.55	*TAC1*[3]	9	23.95	*TAC1*[3]
PH	10	60.19		15	61.26	*D3*[67]; *IPA1* [8]; *d27* [68]
FLL	17	37.09	*NAL1*[11]	28	46.17	
FLW	24	40.73	*NAL1*[11]	33	39.62	*NAL1*[11]
FLLW	14	33.76	*NAL1*[11]	25	39.63	
FLA	4	11.38		8	20.10	
PN	15	37.02	*OsTB1*[69]	16	46.02	*OsTB1*[69]; *te*[70]
PT	12	33.62		10	23.28	
PL	7	10.29		9	23.60	
PC	4	72.19	*Rc*[71]	6	71.67	*Rc*[71]
HC	8	36.30	*Rc*[71]; *Ibf*[72] ^d^	5	38.69	*Ibf*[72] ^d^
Total	126			172		
Average	10.5	34.45		14.3	39.09	

^a^TN, Tiller number; TA, Tiller angle; PH, Plant height; FLL, Flag leaf length; FLW, Flag leaf width; FLLW, The ratio of flag leaf length and flag leaf width; FLA, Flag leaf angle; PN, Panicle number; PT, Panicle type; PL, Panicle length; PC, Pericarp color; HC, Hull color. ^b^ *R* ^*2*^ (%), Total of phenotypic variation for each trait explained by all significant loci; ^c^Details of the known loci are shown in Additional file 11: Table S8 and Additional file 12: Figure S4; ^d^The cause gene has not been confirmed

For tiller number in HZ, the highest SNP peak (seq-rs1206, *P* = 9.89E-06) was detected on chromosome 2, with the SNP explaining ~5.2 % of the total phenotypic variation, and 32 candidate genes were predicted within 200 kb (±100 kb of the peak SNP). For TA, a significant SNP (seq-rs3945) was identified on chromosome 8 both in LS (*P* = 4.52E-04) and HZ (*P* = 1.10E-06), explaining ~3.5 % and ~5.9 % of the total phenotypic variation, respectively. Moreover, 22 candidate genes were contained within 200 kb. For FLL in HZ, a significant locus of seq-rs5454 (*P* = 1.25E-05) was mapped on chromosome 11, explaining ~5.0 % of the total phenotypic variation and 34 candidate genes were obtained. For FLW in HZ, seq-rs4707 (*P* = 3.53E-06) was detected on chromosome 10, explaining ~5.6 % of the total phenotypic variation and 34 candidate genes were predicted. For panicle type (PT) in LS, a peak SNP of seq-rs1802 (*P* = 4.67E-06) was observed on chromosome 3, explaining ~5.4 % of the total phenotypic variation and 38 candidate genes were included. For PC, two significant SNPs, seq-rs1190 and gwseq-rs2, explained ~25.59 % of the phenotypic variation in LS and ~16.74 % in HZ were mapped on chromosomes 2 and 8, respectively. In addition, 29 and 27 candidate genes were predicted (Additional file 11: Table S8). Coincidentally, the two candidate loci were also detected in a previous study using a different *indica* GWAS panel [27]. This demonstrated that the SNP array was capable of capturing the major common variants responsible for morphological traits. Moreover, 16 of 298 (126 plus 172) (~5.37 %) associations were detected across two environments, and 423 candidate genes were predicted in the Rice Genome Annotation Project database (Additional file 13: Table S9).

**Fig. 10:**
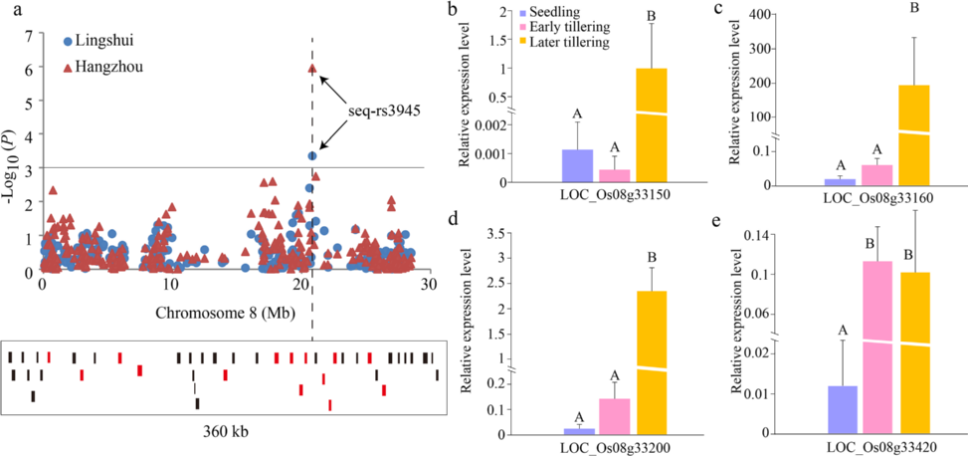
Association mapping and candidate gene expression levels. **a** Manhattan plot on chromosome 8 for tiller angle in two environments and the strongest signal locus, containing 43 candidate genes in a 360-kb genomic region. Red squares represent the genes validated by qPCR. **b**, **c**, **d** and **e** The relative expression levels of LOC_Os08g33150, LOC_Os08g33160, LOC_Os08g33200 and LOC_Os08g33420 in different tillering stages. Different capital letters above the bar plots represent Duncan’s multiple comparison at 0.01 significance level

To verify the effectiveness of our GWAS loci, the new TA locus (seq-rs3945) containing 43 candidate genes (±180 kb, the LD decay distance on chromosome 8) detected in two environments was chosen to test the association (Fig. 10a). Fourteen of the candidate genes with typical gene structures were used to explore gene expression levels in seedling, early and later tillering stages using eight large TA accessions (Additional file 14: Table S10). Most of candidate gene expression levels showed fold changes between two different stages (Additional file 15: Figure S5), especially the expression levels of LOC_Os08g33150 (Fig. 10b), LOC_Os08g33160 (Fig. 10c), LOC_Os08g33200 (Fig. 10d) and LOC_Os08g33420 (Fig. 10e) in the later tillering stage, which were up to 869.9-, 10744.2-, 105.2- and 8.6-fold higher, respectively, than in the seedling stage (Additional file 14: Table S10). The results indicated that the four candidate genes are promising targets participating in the regulation of TA in this region.

### Linkage and pleiotropy

**Fig. 11:**
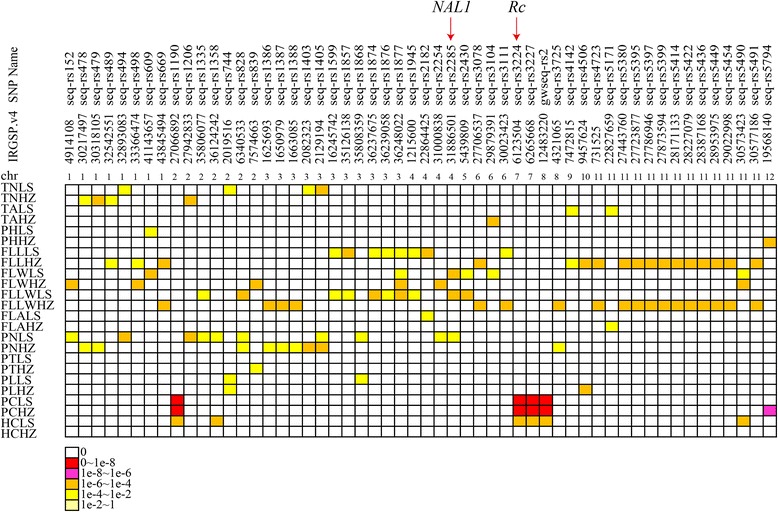
Summary of 54 significant loci across different traits and regions in two environments. Each row represents a trait, and each column represents a marker significantly associated with multiple traits. Different colors shown in the legend correspond to different significance levels. TN, Tiller number; TA, Tiller angle; PH, Plant height; FLL, Flag leaf length; FLW, Flag leaf width; FLLW, The ratio of flag leaf length and flag leaf width; FLA, Flag leaf angle; PN, Panicle number; PT, Panicle type; PL, Panicle length; PC, Pericarp color; HC, Hull color; LS, Lingshui; HZ, Hangzhou

**Fig. 12:**
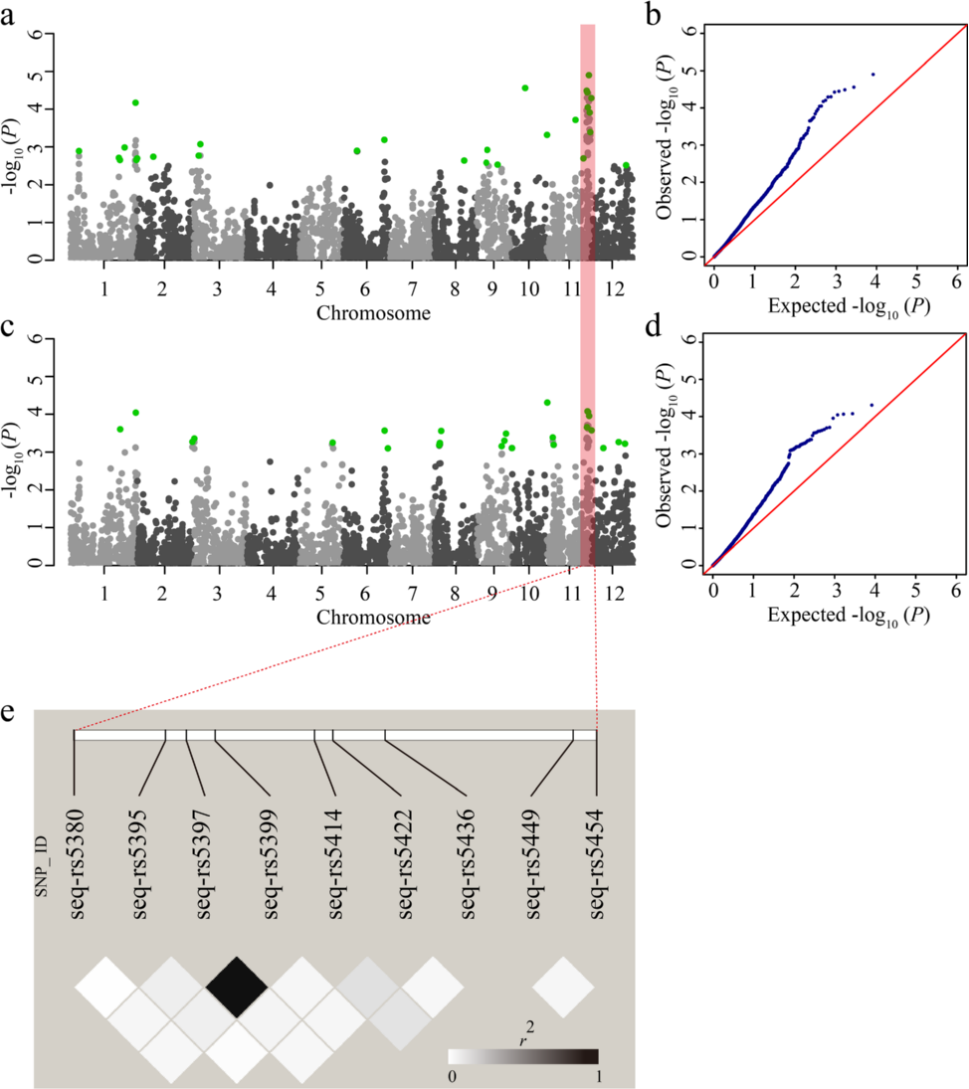
Linkage disequilibrium block of nine loci on chromosome 11 in Hangzhou. **a** Manhattan plots for flag leaf length. **b** Quantile-quantile plots for flag leaf length. **c** Manhattan plots for the ratio of flag leaf length and width. **d** Quantile-quantile plots for the ratio of flag leaf length and width. **e** Linkage disequilibrium analysis of the nine loci. The square lattice panel depicts the extent of LD in the region based on *r* ^2^. The pink range represents the region of a ~1.6 Mb hotspot associated with flag leaf length and the ratio of flag leaf length and width. Green dots represent the peaks of new significant SNPs

Gene linkage and pleiotropy are common phenomena in plant biogenetics. In our study, out of the 456 significant SNPs, 54 known or new candidate SNPs showed clear signal linkage or pleiotropy that was associated with multiple traits in the two environments (Fig. 11). The *NAL1* locus at ~31.8 Mb on chromosome 4 was closely related to FLW, FLLW and PN in LS. Moreover, the correlation coefficients of FLW-FLLW (*r* = −0.60, *P* < 0.001), FLW-PN (*r* = −0.46, *P* < 0.001) and FLLW-PN (*r* = 0.29, *P* < 0.001) were highly significant in LS (Fig. 7a). The *Rc* locus at ~6.1 Mb on chromosome 7 was correlated with both PC and hull color (HC) in LS, and the correlation coefficient was 0.41 (*P* < 0.001) (Fig. 7a). In addition, some new candidate loci having gene linkage or pleiotropy were observed as well. For example, the loci at ~27.1 Mb on chromosome 2 and at ~12.5 Mb on chromosome 8 were highly associated with PC and HC, similar to the *Rc* locus. In particular, a long genome region from ~27.4 to 29.0 Mb on chromosome 11, including nine loci, was associated with both FLL and FLLW in HZ (Fig. 11; Fig. 12a, b, c and d). An LD block analysis of the nine loci showed that these markers had low LD parameter (*r*^2^) values except for markers seq-rs5397 and seq-rs5399 (Fig. 12e). The results demonstrated that a hotspot on chromosome 11 surely existed, not because of the LD block. In conclusion, natural and artificial phenotypic selection may explain gene linkage and pleiotropy in rice domestication.

### Elite alleles and breeding by design

The effects of elite alleles for 10 traits that are directly related to rice plant architecture are summarized in Additional file 16: Table S11). Without considering the effect of interactions among these loci, the more elite alleles were pyramided in varieties, with the larger average phenotypic values increasing significantly (Additional file 17: Figure S6). The results indicated that enhancing the frequency of elite alleles has a significant effect on rice phenotypic improvements. However, some traits, such as TA, flag leaf angle (FLA) and PT, should pyramid more null alleles to obtain lines with erect plant architecture.

Based on the characterization of the new plant type (NPT) [1] and the effects of elite alleles, 37 favorable varieties were chosen as breeding parents to modify the present high-yield plant type (Additional file 18: Table S12). The phenotypes of these favorable varieties closely meet the NPT standards [1], for example, lower tiller (~6 to ~12) and unproductive tiller numbers, erect plant type (TA ≤ 20°), ideal plant height (~80 to ~120 cm), moderate FLL (~20 to ~50 cm), wider FLW (~1.5 to ~2.3 cm), erect leaf and PT, and larger panicles. The number of elite alleles carried by these varieties is listed in Additional file 18: Table S12). In the two environments, most of the elite varieties were different, indicating that morphological traits were highly affected by environment, which was also supported by the ANOVA (Table 2). Moreover, in different environments, the rice plant type modification depended on different parents and combinations. However, two elite accessions (Zaoxiang17 and Lvyuanzhan) were found repeatedly in two environments, indicating that the two varieties possess unique phenotypes and ideal numbers of elite alleles.

## Discussion

Rice is a staple food and provides energy for more than half of the world’s population. In recent decades, rice production has only increased slightly and improving rice yields has become a pressing problem. Plant type modification is a potential way to solve these challenges. The NPT was characterized by Khush et al. in the 1990s to change rice plant architecture to enhance rice production [1]. In this study, a GWAS was performed using a larger natural population than in previous studies [27, 28]. These materials originated from 25 countries, which represented all of the major rice-growing regions in the world (Fig. 1; Additional file 1: Table S1). In addition, 12 morphological traits were systematically measured in two different environments. This work can comprehensively reveal the genetic architecture of morphological traits and will provide elite parents for rice plant type modifications.

Although GWAS is a feasible tool to reveal complicated genetic variations in rice, it may be highly affected by population structure, resulting in false positive associations [26]. Structural association and genomic control are two common strategies performed in human and plant association mapping to reduce the rate of false positive loci [50, 57–59]. In our studies, the association panel only included *indica* subspecies to control false positive associations. In addition, the program STRUCTURE was used to uncover population structures in the *indica* panel for a further GWAS analysis [40, 41]. Four subpopulations (POP1 to POP4) and a mixed group were obtained and subsequently supported by a PCA, which was consider to be another fast and effectively inferred population structure [60] (Fig. 4; Additional file 6: Table S5). Because of domestication and artificial selection, genetic relatedness among individuals, which generated numerous spurious associations, is inevitable in the association panel [50]. In this work, familial relationships were estimated using SPAGeDi, which is a versatile program that analyzes spatial genetic structures [45]. About 83 % of the kinship coefficient values were less than 0.01 (Fig. 5), indicating that there was no or weak relatedness between pairwise varieties in the panel (Additional file 8: Figure S3).

As mentioned above, population structure (Q-matrix) and relative kinship (K matrix) play key roles in controlling type I error. Therefore, combining the two factors has been suggested to reduce the false positive loci in association mapping [61, 62]. In this study, four models with different combinations of K and Q were performed for all of the traits in the two environments. The Q + K model was regarded as the best model to reduce the rate of false positive associations (Figs. 8, 9). These results were also supported by an earlier study in maize [61]. Consequently, the Q + K model was considered to be the ideal choice for our association mapping.

LD plays an extremely important role in association mapping and determines the resolution of an association study [63]. In our study, the LD decay distance varied over 12 different chromosomes, ranging from ~96.15 kb on chromosome 5 to ~421.39 kb on chromosome 7 (Additional file 9: Table S6). This may be due to the differences in the recombination rates on the 12 chromosomes. Moreover, the whole genome-wide and the average LD decay distances were 109.37 kb and 214.69 kb, respectively (Additional file 9: Table S6). This is in agreement with the previous calculation [27, 56] but much larger than maize (~1 to 10 kb) [64]. These results indicated that LD extends over a much longer distance in self-pollinated species, such as rice [27], soybean [35] and *Arabidopsis* [65], than in cross-pollinated crops, such as maize [64]. Marker density is another factor in the power of the association analysis. Taking the rice LD decay rate into consideration, even though our average marker density is ~1 SNP per 100 kb in the whole genome (Fig. 2), which is lower than in previous studies [27, 28, 31], we also expected to have reasonable power to detect common genomic large-effect variations in our study.

To date, many morphological genes (or QTLs) have been identified in rice using the bi-parental mapping strategy [2–12, 15–22]. Compared with linkage mapping, the GWAS method not only provides a higher mapping resolution but also enables the detection of a greater number of candidate loci [27, 30, 36]. Hence, GWAS has been widely performed to reveal the genetic variation of complex traits in rice [27–32], maize [33, 34], soybean [35, 36] and wheat [37]. To better understand the genetic variation of morphologic traits in rice, GWAS was conducted for 12 traits in two environments. A total of 298 significant loci, including both reported and new loci, were identified (Table 4; Additional file 11: Table S8; Additional file 12: Figure S4), and 16 (~5.37 %) loci were detected across the two environments (Additional file 13: Table S9). Morphological traits are highly affected by environment, which was supported by the ANOVA (Table 2) and a previous study [66]. For the new loci detected in the two environments, 423 candidate genes were obtained within a 200-kb genomic region (±100 kb of the peak SNP) in the Rice Genome Annotation Project database (Additional file 13: Table S9), and the functional variants, together with the effects of these genes, will be tested and validated using genetic transformation in the future. Moreover, the known loci that are directly related to rice plant morphology, such as *sd1* [7], *MOC1* [2], *D53* [9] and *LC1* [15], were not detected in our work. One possible reason was the insufficient SNP coverage in some genomic regions, especially near the centromere. Another possible explanation was that some genetic variations were actually rare in the *indica* panel. The third possible reason was false negative associations because of the strict threshold value.

In our study, we observed that some loci were significantly associated with multiple traits in the same or different environments. The typical example was the *Rc* gene at ~6.1 Mb on chromosome 7, which was highly associated with both PC and HC traits in LS (Fig. 11), supported by a correlation coefficient value between the two traits (*r* = 0.41, *P* < 0.001) (Fig. 7a). Previous reports showed that *NAL1* had pleiotropic effects on leaf anatomy, photosynthesis rate, spikelet number and grain yield [12–14]. Our results indicated that *NAL1* was associated with FLW, FLLW and PN in LS (Fig. 11), and the correlation coefficient values of FLW-FLLW, FLW-PN and FLLW-PN were also significant (Fig. 7a). In addition, an extensive region (~1.6 Mb on chromosome 11) described as ‘mountain range’ [26], which was associated with FLL and FLLW, was identified in HZ (Fig. 11; Additional file 12: Figure S4). That this was caused by several genes (or QTLs) that contribute to the two traits was confirmed by the LD block analysis (Fig. 12). This result demonstrated that the ‘mountain range’ may be critical for rice leaf architecture and was maintained during rice domestication.

The results of the allelic analysis demonstrated that with the continual pyramid of elite alleles, the average phenotypic value increases significantly (Additional file 17: Figure S6), and these elite alleles will be beneficial to rice improvement in the future. Based on the characterization of NPT [1] and the elite alleles (Additional file 16: Table S11), 37 elite varieties were obtained as breeding parents to modify the plant architecture. Furthermore, rice morphological traits were highly affected by the environments (Table 2), and most of these traits were significantly correlated with each other (Fig. 7). Hence, rice plant type modification depends not only on the pyramiding of elite alleles but also on the effects of the local environment. More importantly, the interactions among these loci should also not be ignored.

## Conclusions

In this work, we identified a number of significant marker-trait associations and candidate genes, and also uncovered the genetic variations of rice morphological traits using the GWAS strategy in 469 *indica* accessions. Moreover, 16 significant loci, including 423 candidate genes, were obtained across two environments. Relative expression analyses identified four candidate genes as the most promising regulators of tiller angle. In addition, many significant loci showed pleiotropy and gene linkage. A long genomic region covering ~1.6 Mb may be critical for rice leaf architecture. The genetic variants and elite alleles will be useful for rice improvement. Furthermore, the biological validation of the candidate genes will be focused on in future research. Genome selection and breeding by design using the elite varieties as parents will also be of interest in future.

### Availability of supporting data

The 5291 SNPs genotype data for the 523 accessions was deposited in the Dryad digital repository: http://dx.doi.org/10.5061/dryad.cp25h.

## Additional files

Additional file 1: Table S1. Origination and classification of 523 germplasm accessions. (XLSX 41 kb) Additional file 2: Figure S1. Methods of evaluating tiller angle, flag leaf angle and panicle type. (PDF 94 kb) Additional file 3: Table S2. Information on the 4,136 SNPs. (XLSX 567 kb) Additional file 4: Table S3. The population differentiation statistics (*F*_ST_) between pairwise subpopulations. (PDF 9 kb) Additional file 5: Table S4. Summary of primers used for quantitative real-time PCR. (XLSX 15 kb) Additional file 6: Table S5. Subpopulations and genetic components of 469 *indica* accessions. (XLSX 44 kb) Additional file 7: Figure S2. The geographic distribution of different subpopulations. (PDF 71 kb) Additional file 8: Figure S3. Heatmap of pairwise relative kinship values. (PDF 242 kb) Additional file 9: Table S6. Linkage disequilibrium (LD) decay distance of the 12 chromosomes. (PDF 13 kb) Additional file 10: Table S7. Phenotypic data for 12 traits in Lingshui and Hangzhou. (XLSX 116 kb) Additional file 11:Table S8. Summary of 298 significant peak SNPs, including known genes (or QTLs) and new candidate genes. (XLSX 109 kb) Additional file 12: Figure S4. Manhattan and Quantile-Quantile plots of GWAS for all 12 traits in two environments. (PDF 1926 kb) Additional file 13: Table S9. Summary of the 16 loci detected in two environments and the 423 candidate genes. (XLSX 25 kb) Additional file 14: Table S10. Relative expression levels of 14 tiller angle candidate genes at different tillering stages. (XLSX 27 kb) Additional file 15: Figure S5. Fold changes of 14 tiller angle candidate genes in different tillering stages. (PDF 97 kb) Additional file 16: Table S11. Summary of elite alleles and phenotypic effects. (XLSX 30 kb) Additional file 17: Figure S6. Pyramid effect analysis for different numbers of elite alleles. (PDF 464 kb) Additional file 18: Table S12. Summary of the number of elite alleles carried by each favorable accession. (XLSX 19 kb)

## Abbreviations

E: Environment
FLA: Flag leaf angle
FLL: Flag leaf length
FLLW: The ratio of flag leaf length and flag leaf width
FLW: Flag leaf width
G: Genotype
GWAS: Genome-wide association study
HC: Hull color
HZ: Hangzhou
LD: Linkage disequilibrium
LS: Lingshui
PC: Pericarp color
PCA: Principal component analysis
PH: Plant height
PL: Panicle length
PN: Panicle number
PT: Panicle type
QTL: Quantitative trait locus
SE: Standard error
SNP: Single nucleotide polymorphism
TA: Tiller angle
TN: Tiller number

## References

[1] Khush GS (1995). Breaking the Yield Frontier of Rice. Geo J.

[2] Li XY, Qian Q, Fu ZM, Wang YH, Xiong GS, Zeng DL (2003). Control of tillering in rice. Nature.

[3] Yu BS, Lin ZW, Li HX, Li XJ, Li JY, Wang YH (2007). *TAC1*, a major quantitative trait locus controlling tiller angle in rice. Plant J.

[4] Li PJ, Wang YH, Qian Q, Fu ZM, Wang M, Zeng DL (2007). *LAZY1* controls rice shoot gravitropism through regulating polar auxin transport. Cell Res.

[5] Jin J, Huang W, Gao JP, Yang J, Shi M, Zhu MZ (2008). Genetic control of rice plant architecture under domestication. Nat Genet.

[6] Tan LB, Li XR, Liu FX, Sun XY, Li CG, Zhu ZF (2008). Control of a key transition from prostrate to erect growth in rice domestication. Nat Genet.

[7] Sasaki A, Ashikari M, Ueguchi-Tanaka M, Itoh H, Nishimura A, Swapan D (2002). Green revolution: a mutant gibberellin-synthesis gene in rice. Nature.

[8] Jiao YQ, Wang YH, Xue DW, Wang J, Yan MX, Liu GF (2010). Regulation of *OsSPL14* by OsmiR156 defines ideal plant architecture in rice. Nat Genet.

[9] Jiang L, Liu X, Xiong GS, Liu HH, Chen FL, Wang L (2013). DWARF 53 acts as a repressor of strigolactone signalling in rice. Nature.

[10] Zhou F, Lin QB, Zhu LH, Ren YL, Zhou KN, Shabek N (2013). D14-SCF^D3^-dependent degradation of D53 regulates strigolactone signalling. Nature.

[11] Qi J, Qian Q, Bu QY, Li SY, Chen Q, Sun JQ (2008). Mutation of the Rice *Narrow leaf1* Gene, Which Encodes a Novel Protein, Affects Vein Patterning and Polar Auxin Transport. Plant Physiol.

[12] Chen ML, Luo J, Shao GN, Wei XJ, Tang SQ, Sheng ZH (2012). Fine mapping of a major QTL for flag leaf width in rice, *qFLW4*, which might be caused by alternative splicing of *NAL1*. Plant Cell Rep.

[13] Fujita D, Trijatmiko KR, Tagle AG, Sapasap MV, Koide Y, Sasaki K (2013). *NAL1* allele from a rice landrace greatly increases yield in modern *indica* cultivars. Proc Natl Acad Sci U S A.

[14] Takai T, Adachi S, Taguchi-Shiobara F, Sanoh-Arai Y, Iwasawa N, Yoshinaga S (2013). A natural variant of *NAL1*, selected in high-yield rice breeding programs, pleiotropically increases photosynthesis rate. Sci Rep-UK.

[15] Zhao SQ, Xiang JJ, Xue HW (2013). Studies on the Rice LEAF INCLINATION1 (LC1), an IAA-amido Synthetase, Reveal the Effects of Auxin in Leaf Inclination Control. Mol Plant.

[16] Shen B, Yu WD, Zhu YJ, Fan YY, Zhuang JY (2012). Fine mapping of a major quantitative trait locus, *qFLL6.2*, controlling flag leaf length and yield traits in rice (*Oryza sativa* L.). Euphytica.

[17] Wang P, Zhou GL, Yu HH, Yu SB (2011). Fine mapping a major QTL for flag leaf size and yield-related traits in rice. Theor Appl Genet.

[18] Zhang B, Ye WJ, Ren DY, Tian P, Peng YL, Gao Y (2015). Genetic analysis of flag leaf size and candidate genes determination of a major QTL for flag leaf width in rice. Rice.

[19] Zhang GH, Li SY, Wang L, Ye WJ, Zeng DL, Rao YC (2014). *LSCHL4* from *Japonica* Cultivar, Which Is Allelic to *NAL1*, Increases Yield of *Indica* Super Rice 93–11. Mol Plant.

[20] Yan CJ, Zhou JH, Yan S, Chen F, Yeboah M, Tang SZ (2007). Identification and characterization of a major QTL responsible for erect panicle trait in japonica rice (*Oryza sativa* L.). Theor Appl Genet.

[21] Zhu KM, Tang D, Yan CJ, Chi ZC, Yu HX, Chen JM (2010). *ERECT PANICLE2* Encodes a Novel Protein That Regulates Panicle Erectness in *Indica* Rice. Genetics.

[22] Li SB, Qian Q, Fu ZM, Zeng DL, Meng XB, Kyozuka J (2009). *Short panicle1* encodes a putative PTR family transporter and determines rice panicle size. Plant J.

[23] Zhu CS, Gore M, Buckler ES, Yu JM (2008). Status and Prospects of Association Mapping in Plants. Plant Genome.

[24] Edwards AO, Ritter R, Abel KJ, Abel KJ, Manning A, Panhuysen C (2005). Complement Factor H Polymorphism and Age-Related Macular Degeneration. Science.

[25] Duijvesteijn N, Knol EF, Merks JWM, Crooijmans RPMA, Groenen MAM, Bovenhuis H (2010). A genome-wide association study on androstenone levels in pigs reveals a cluster of candidate genes on chromosome 6. BMC Genet.

[26] Atwell S, Huang YS, Vilhjálmsson BJ, Willems G, Horton M, Li Y (2010). Genome-wide association study of 107 phenotypes in *Arabidopsis thaliana* inbred lines. Nature.

[27] Huang XH, Wei XH, Sang T, Zhao Q, Feng Q, Zhao Y (2010). Genome-wide association studies of 14 agronomic traits in rice landraces. Nat Genet.

[28] Zhao KY, Tung CW, Eizenga GC, Wright MH, Ali ML, Price AH (2011). Genome-wide association mapping reveals a rich genetic architecture of complex traits in *Oryza sativa*. Nat Commun.

[29] Huang XH, Zhao Y, Wei XH, Li CY, Wang AH, Zhao Q (2011). Genome-wide association study of flowering time and grain yield traits in a worldwide collection of rice germplasm. Nat Genet.

[30] Wang CH, Yang YL, Yuan XP, Xu Q, Feng Y, Yu HY (2014). Genome-wide association study of blast resistance in indica rice. BMC Plant Biol.

[31] Wang QX, Xie WB, Xing HK, Yan J, Meng XZ, Li XL (2015). Genetic architecture of natural variation in rice chlorophyll content revealed by genome wide association study. Mol Plant.

[32] Kumar V, Singh A, Mithra SVA, Krishnamurthy SL, Parida SK, Jain S (2015). Genome-wide association mapping of salinity tolerance in rice (*Oryza sativa*). DNA Res.

[33] Tian F, Bradbury PJ, Brown PJ, Hung H, Sun Q, Flint-Garcia S (2011). Genome-wide association study of leaf architecture in the maize nested association mapping population. Nat Genet.

[34] Riedelsheimer C, Lisec J, Czedik-Eysenberg A, Sulpice R, Flis A, Grieder C (2012). Genome-wide association mapping of leaf metabolic profiles for dissecting complex traits in maize. Proc Natl Acad Sci U S A.

[35] Wen ZX, Tan RJ, Yuan JZ, Bales C, Du WY, Zhang SC (2014). Genome-wide association mapping of quantitative resistance to sudden death syndrome in soybean. BMC Genomics.

[36] Zhang JP, Song QJ, Cregan PB, Nelson RL, Wang XZ, Wu JX (2015). Genome-wide association study for flowering time, maturity dates and plant height in early maturing soybean (*Glycine max*) germplasm. BMC Genomics.

[37] Sukumaran S, Dreisigacker S, Lopes M, Chavez P, Reynolds MP (2015). Genome-wide association study for grain yield and related traits in an elite spring wheat population grown in temperate irrigated environments. Theor Appl Genet.

[38] Huang XH, Han B (2014). Natural Variations and Genome-Wide Association Studies in Crop Plants. Annu Rev Plant Biol..

[39] Liu KJ, Muse SV (2005). PowerMarker: an integrated analysis environment for genetic marker analysis. Bioinformatics.

[40] Pritchard JK, Stephens M, Donnelly P (2000). Inference of Population Structure Using Multilocus Genotype Data. Genetics.

[41] Evanno G, Regnaut S, Goudet J (2005). Detecting the number of clusters of individuals using the software STRUCTURE: a simulation study. Mol Ecol.

[42] Jakobsson M, Rosenberg NA (2007). CLUMPP: a cluster matching and permutation program for dealing with label switching and multimodality in analysis of population structure. Bioinformatics.

[43] Nei M (1972). Genetic distance between populations. Am Nat.

[44] Rohlf F (2000). Numerical Taxonomy and Multivariate Analysis System.

[45] Hardy OJ, Vekemans X (2002). SPAGeDi: a versatile computer program to analyse spatial genetic structure at the individual or population levels. Mol Ecol Notes.

[46] Bradbury PJ, Zhang ZW, Kroon DE, Casstevens TM, Ramdoss Y, Buckler ES (2007). TASSEL: software for association mapping of complex traits in diverse samples. Bioinformatics.

[47] Barrett JC, Fry B, Maller J, Daly MJ (2005). Haploview: analysis and visualization of LD and haplotype maps. Bioinformatics.

[48] Kang HM, Zaitlen NA, Wade CM, Kirby A, Heckerman D, Daly MJ (2008). Efficient Control of Population Structure in Model Organism Association Mapping. Genetics.

[49] Zhang ZW, Ersoz E, Lai CQ, Todhunter RJ, Tiwari HK, Gore MA (2010). Mixed linear model approach adapted for genome-wide association studies. Nat Genet.

[50] Yu JM, Pressoir G, Briggs WH, Bi IV, Yamasaki M, Doebley JF (2005). A unified mixed-model method for association mapping that accounts for multiple levels of relatedness. Nat Genet.

[51] Zhang ZW, Buckler ES, Casstevens TM, Bradbury PJ (2009). Software engineering the mixed model for genome-wide association studies on large samples. Brief Bioinform.

[52] Goodman SN (2001). Of *P*-Values and Bayes: A Modest Proposal. Epidemiology.

[53] Katki HA (2008). Invited Commentary: Evidence-based Evaluation of *p* Values and Bayes Factors. Am J Epidemiol.

[54] Moran MD (2003). Arguments for rejecting the sequential Bonferroni in ecological studies. Oikos.

[55] Yang N, Lu YL, Yang XH, Huang J, Zhou Y, Ali F (2014). Genome Wide Association Studies Using a New Nonparametric Model Reveal the Genetic Architecture of 17 Agronomic Traits in an Enlarged Maize Association Panel. PLoS ONE.

[56] Mather KA, Caicedo AL, Polato NR, Olsen KM, McCouch S, Purugganan MD (2007). The Extent of Linkage Disequilibrium in Rice (*Oryza sativa* L.). Genetics.

[57] Pritchard JK, Stephens M, Rosenberg NA, Donnelly P (2000). Association Mapping in Structured Populations. Am J Hum Genet.

[58] Devlin B, Roeder K (1999). Genomic Control for Association Studies. Biometrics.

[59] Marchini J, Cardon LR, Phillips MS, Donnelly P (2004). The effects of human population structure on large genetic association studies. Nat Genet.

[60] Price AL, Patterson NJ, Plenge RM, Weinblatt ME, Shadick NA, Reich D (2006). Principal components analysis corrects for stratification in genome-wide association studies. Nat Genet.

[61] Yang XH, Yan JB, Shah T, Warburton ML, Li Q, Li L (2010). Genetic analysis and characterization of a new maize association mapping panel for quantitative trait loci dissection. Theor Appl Genet.

[62] Yang XH, Gao SB, Xu ST, Zhang ZX, Prasanna BM, Li L (2011). Characterization of a global germplasm collection and its potential utilization for analysis of complex quantitative traits in maize. Mol Breeding.

[63] Flint-Garcia SA, Thornsberry JM, Buckler ES (2003). Structure of linkage disequilibrium in plants. Annu Rev Plant Biol.

[64] Yan JB, Shah T, Warburton ML, Buckler ES, McMullen MD, Crouch J (2009). Genetic Characterization and Linkage Disequilibrium Estimation of a Global Maize Collection Using SNP Markers. PLoS ONE.

[65] Nordborg M, Borevitz JO, Bergelson J, Berry CC, Chory J, Hagenblad J (2002). The extent of linkage disequilibrium in Arabidopsis *thaliana*. Nat Genet.

[66] Xu FF, Tang FF, Shao YF, Chen YL, Tong C, BAO JS (2014). Genotype × Environment Interactions for Agronomic Traits of Rice Revealed by Association Mapping. Rice Sci.

[67] Zhao JF, Wang T, Wang MX, Liu YY, Yuan SJ, Gao YA (2014). DWARF3 Participates in an SCF Complex and Associates with DWARF14 to Suppress Rice Shoot Branching. Plant Cell Physiol.

[68] Lin H, Wang RX, Qian Q, Yan MX, Meng XB, Fu ZM (2009). DWARF27, an Iron-Containing Protein Required for the Biosynthesis of Strigolactones, Regulates Rice Tiller Bud Outgrowth. Plant Cell.

[69] Takeda T, Suwa Y, Suzuki M, Kitano H, Ueguchi-Tanaka M, Ashikari M (2003). The *OsTB1* gene negatively regulates lateral branching in rice. Plant J.

[70] Lin QB, Wang D, Dong H, Gu SH, Cheng ZJ, Gong J (2012). Rice APC/C^TE^ controls tillering by mediating the degradation of *MONOCULM 1*. Nat Commun.

[71] Sweeney MT, Thomson MJ, Pfeil BE, McCouch S (2006). Caught Red-Handed: *Rc* Encodes a Basic Helix-Loop-Helix Protein Conditioning Red Pericarp in Rice. Plant Cell.

[72] Cui JJ, Fan SC, Shao T, Huang ZJ, Zheng DL, Tang D (2007). Characterization and Fine Mapping of the *ibf* Mutant in Rice. J Integr Plant Biol.

