# Supplementary material for: Genetic variation and association mapping for 12 agronomic traits in indica rice
Source: BMC Genomics. 2015 Dec 16;16:1067. doi: 10.1186/s12864-015-2245-2 (PMC4681178; doi:10.1186/s12864-015-2245-2)
Supplement: Additional file 2: Figure S1. — Methods of evaluating tiller angle, flag leaf angle and panicle type. (PDF 94 kb) [file 12864_2015_2245_MOESM2_ESM.pdf]

Methods of evaluating 12 morphological traits:

Tiller number (TN): count the number of tillers at the booting stage;

Tiller angle (TA): measure the tiller angle between vertical and marginal tiller at the tillering stage (Figure S1a);

Plant height (PH): measure the height of plant from the soil surface to the top of main panicle at the maturation stage;

Flag leaf length (FLL): measure the length of flag leaf at the maturation stage;

Flag leaf width (FLW): measure the width of flag leaf at the maturation stage;

Ratio of flag leaf length and width (FLLW): flag leaf length / flag leaf width;

Flag leaf angle (FLA): estimate the flag leaf angle at the maturation stage (Figure S1b);

Panicle number (PN): count the number of grain panicle at the maturation stage;

Panicle type (PT): estimate the panicle type at the heading stage (Figure S1c);

Panicle length (PL): measure the length of main panicle (without hull length) at the maturation stage;

Pericarp color (PC): describe the color of unpolished rice grain, white is coded as 0 and other colors are coded as 1;

Hull color (HC): describe the color of seed hull, gold yellow is coded as 0 and other colors are coded as 1;

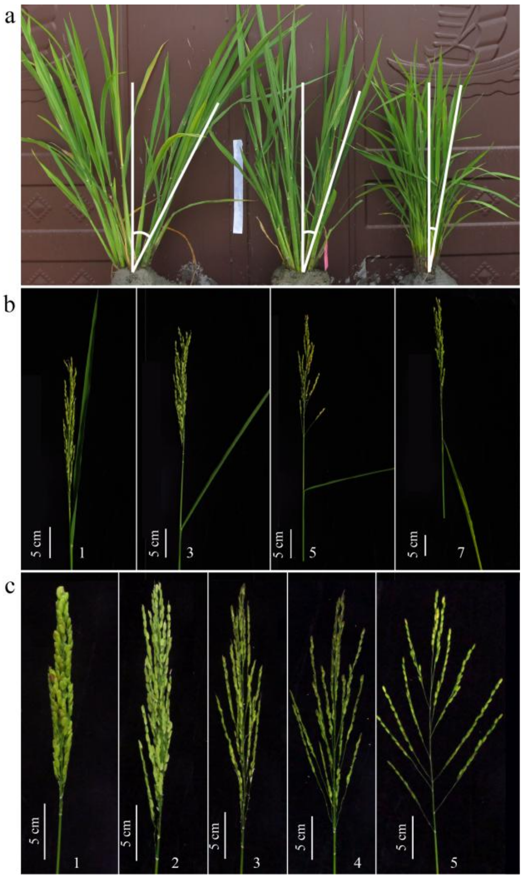

Figure S1. Methods of evaluating tiller angle, flag leaf angle and panicle type. (a) Tiller angle, bar = 20 cm; (b) Flag leaf angle, code 1 to 7 represent erect leaf type to droop leaf type; (c) Panicle type, code 1 to 5 represent compact panicle type to loose panicle type.
