# Supplementary material for: Genetic variation and association mapping for 12 agronomic traits in indica rice
Source: BMC Genomics. 2015 Dec 16;16:1067. doi: 10.1186/s12864-015-2245-2 (PMC4681178; doi:10.1186/s12864-015-2245-2)
Supplement: Additional file 4: Table S3. — The population differentiation statistics (F ST) between pairwise subpopulations. (PDF 9 kb) [file 12864_2015_2245_MOESM4_ESM.pdf]

Table S3. The population differentiation statistics (F_{ST}) between pairwise subpopulations.

523 whole panel			469 *indica* panel			
	japonica		POP1	POP2	POP3	POP4
indica	0.56	POP2	0.24			
		POP3	0.24	0.23		
		POP4	0.25	0.18	0.22	
		Mixed	0.13	0.10	0.05	0.07
