# Supplementary material for: Genetic variation and association mapping for 12 agronomic traits in indica rice
Source: BMC Genomics. 2015 Dec 16;16:1067. doi: 10.1186/s12864-015-2245-2 (PMC4681178; doi:10.1186/s12864-015-2245-2)
Supplement: Additional file 7: Figure S2. — The geographic distribution of different subpopulations. (PDF 71 kb) [file 12864_2015_2245_MOESM7_ESM.pdf]

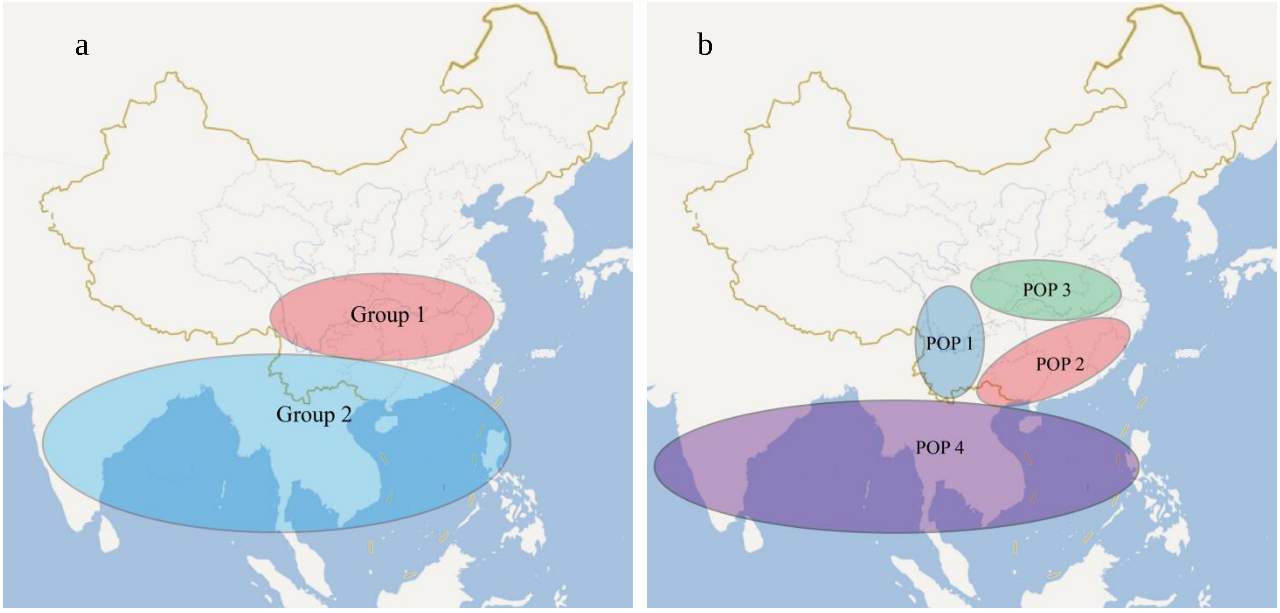

K = 2 K = 4

Figure S2 The geographic distribution of different subpopulations. (a) Two groups were inferred by STRUCTURE, K = 2; (b) Four subpopulations were inferred by STRUCTURE, K = 4.
