# Supplementary material for: Genetic variation and association mapping for 12 agronomic traits in indica rice
Source: BMC Genomics. 2015 Dec 16;16:1067. doi: 10.1186/s12864-015-2245-2 (PMC4681178; doi:10.1186/s12864-015-2245-2)
Supplement: Additional file 8: Figure S3. — Heatmap of pairwise relative kinship values. (PDF 242 kb) [file 12864_2015_2245_MOESM8_ESM.pdf]

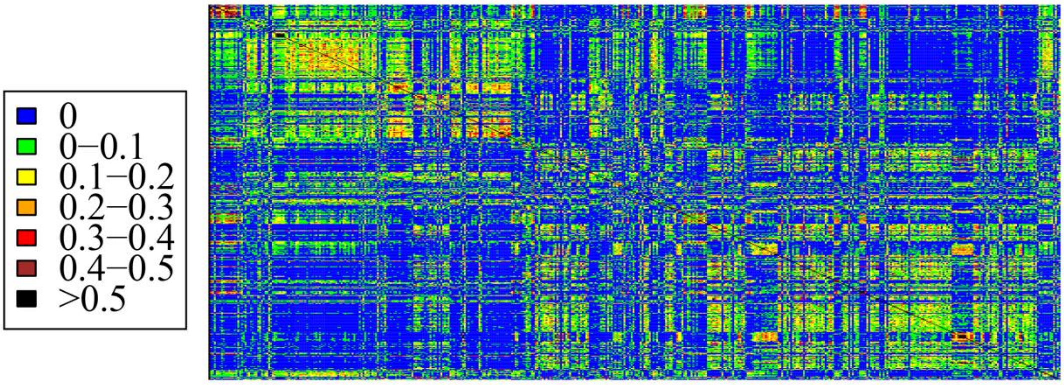

Figure S3. Heatmap of pairwise relative kinship values. Individuals are ordered according 469 *indica* accession ID and each pixel in the square indicates the range of kinship value as shown in the left colored legend.
