# Supplementary material for: Genetic variation and association mapping for 12 agronomic traits in indica rice
Source: BMC Genomics. 2015 Dec 16;16:1067. doi: 10.1186/s12864-015-2245-2 (PMC4681178; doi:10.1186/s12864-015-2245-2)
Supplement: Additional file 9: Table S6. — Linkage disequilibrium (LD) decay distance of the 12 chromosomes. (PDF 13 kb) [file 12864_2015_2245_MOESM9_ESM.pdf]

Table S6. Linkage disequilibrium (LD) decay distance of the 12 chromosomes.

Chromosome	Regression equation	R^2 [a]	r^2_{max} [b]	LD decay distance [c]
1	y = -0.091ln(x) + 0.693	0.8272	0.5503	98.67
2	y = -0.11ln(x) + 0.7738	0.9279	0.4891	122.90
3	y = -0.115ln(x) + 0.8756	0.8257	0.5640	174.47
4	y = -0.079ln(x) + 0.6695	0.7210	0.5581	140.11
5	y = -0.113ln(x) + 0.8267	0.8157	0.6215	96.15
6	y = -0.087ln(x) + 0.7311	0.8777	0.4810	281.20
7	y = -0.062ln(x) + 0.6146	0.5639	0.4798	421.39
8	y = -0.085ln(x) + 0.6495	0.8616	0.4157	180.53
9	y = -0.066ln(x) + 0.5911	0.6425	0.4595	238.65
10	y = -0.09ln(x) + 0.7435	0.7417	0.4597	301.03
11	y = -0.094ln(x) + 0.7203	0.9020	0.5078	142.84
12	y = -0.084ln(x) + 0.7233	0.6779	0.4494	378.31
Average				214.69
Whole panel	y = -0.133ln(x) + 0.9733	0.9422	0.6978	109.37

[a] coefficient of determination; [b] the maximum of correlation coefficient; [c] the distance (kb) is at which the r^2 dropped to half of the r^2_{max}.
