# Supplementary material for: Genetic variation and association mapping for 12 agronomic traits in indica rice
Source: BMC Genomics. 2015 Dec 16;16:1067. doi: 10.1186/s12864-015-2245-2 (PMC4681178; doi:10.1186/s12864-015-2245-2)
Supplement: Additional file 12: Figure S4. — Manhattan and Quantile-Quantile plots of GWAS for all 12 traits in two environments. (PDF 1926 kb) [file 12864_2015_2245_MOESM12_ESM.pdf]

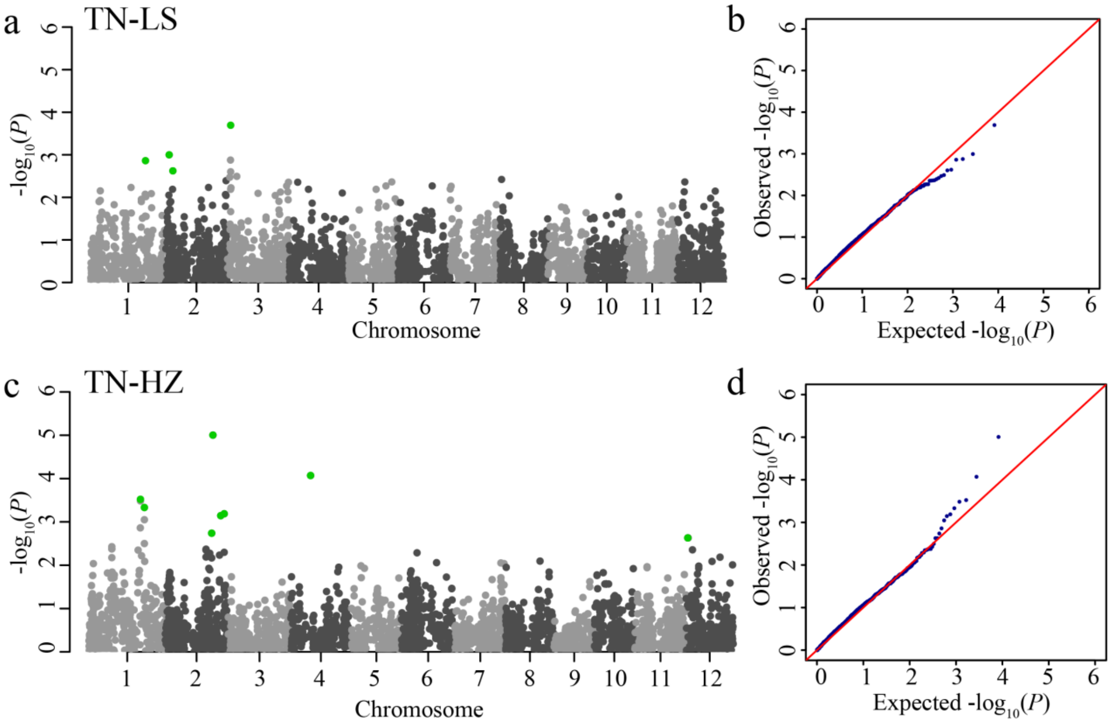

a
TN-LS
-log10(P)
Chromosome
1 2 3 4 5 6 7 8 9 10 11 12
b
Observed -log10(P)
Expected -log10(P)
c
TN-HZ
-log10(P)
Chromosome
1 2 3 4 5 6 7 8 9 10 11 12
d
Observed -log10(P)
Expected -log10(P)

(Continued)

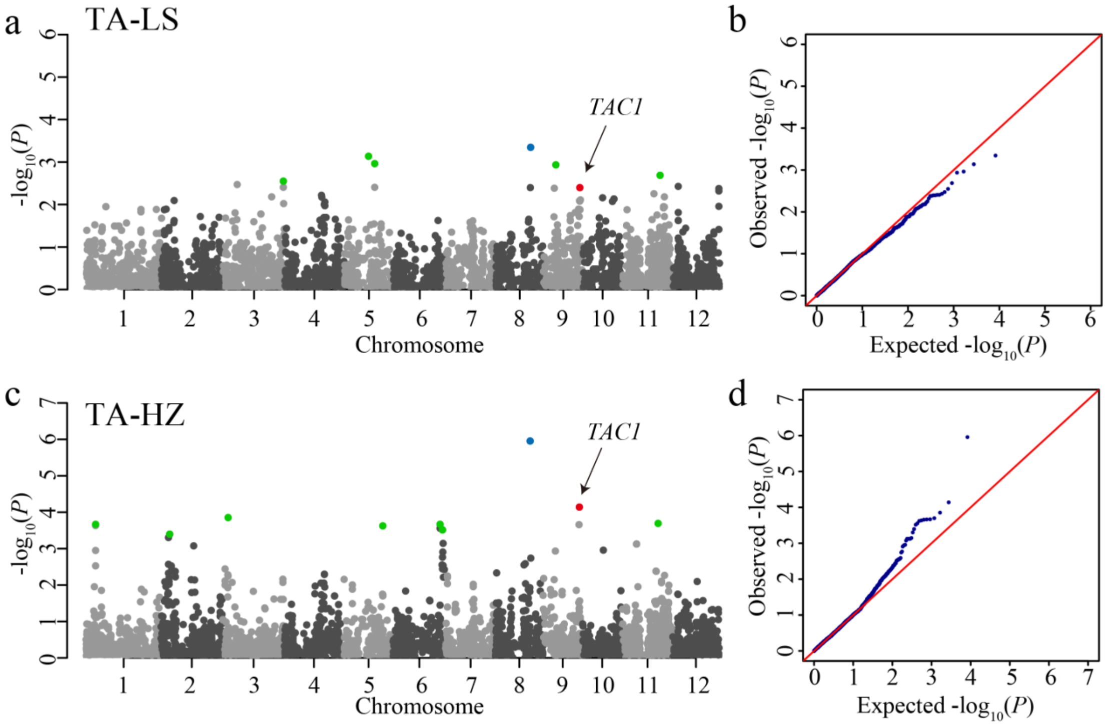
a
TA-LS
-log10(P)
TAC1
Chromosome
b
Observed -log10(P)
Expected -log10(P)
c
TA-HZ
-log10(P)
TAC1
Chromosome
d
Observed -log10(P)
Expected -log10(P)

(Continued)

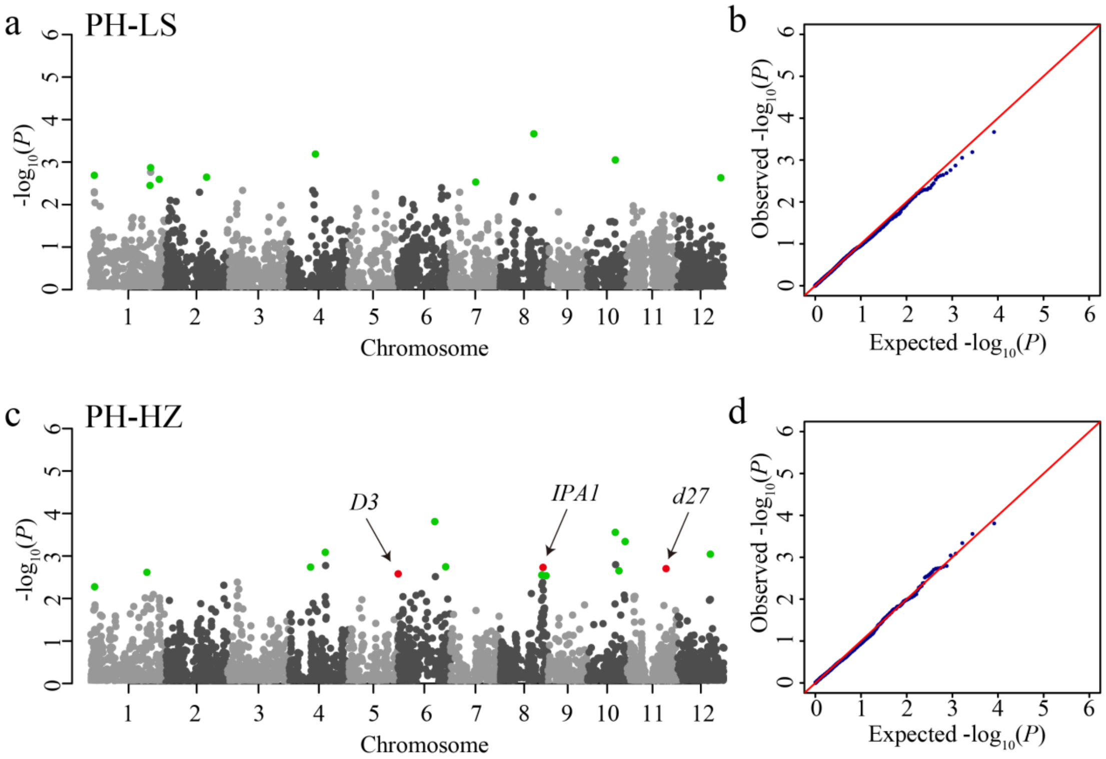

a PH-LS
-log10(P)
Chromosome
b
Observed -log10(P)
Expected -log10(P)
c PH-HZ
D3
IPA1
d27
-log10(P)
Chromosome
d
Observed -log10(P)
Expected -log10(P)

(Continued)

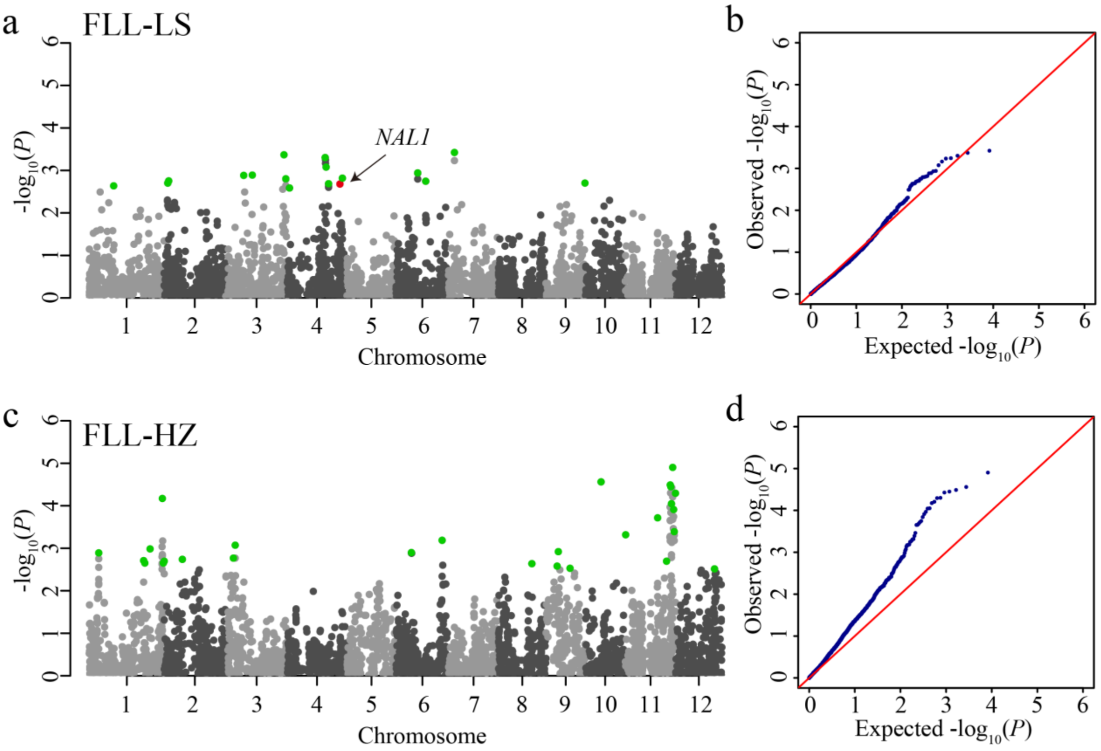

a
FLL-LS
NAL1
-log10(P)
Chromosome
b
Observed -log10(P)
Expected -log10(P)
c
FLL-HZ
-log10(P)
Chromosome
d
Observed -log10(P)
Expected -log10(P)

(Continued)

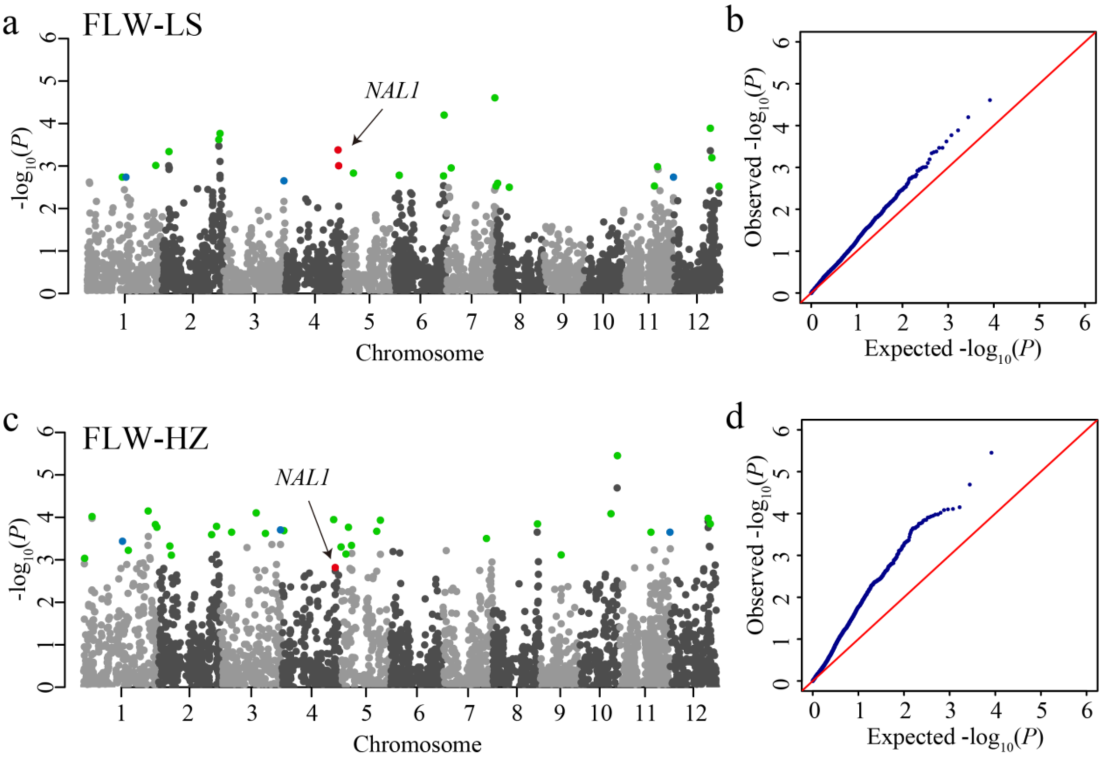

a
FLW-LS
NAL1
-log10(P)
Chromosome
b
Observed -log10(P)
Expected -log10(P)
c
FLW-HZ
NAL1
-log10(P)
Chromosome
d
Observed -log10(P)
Expected -log10(P)

(Continued)

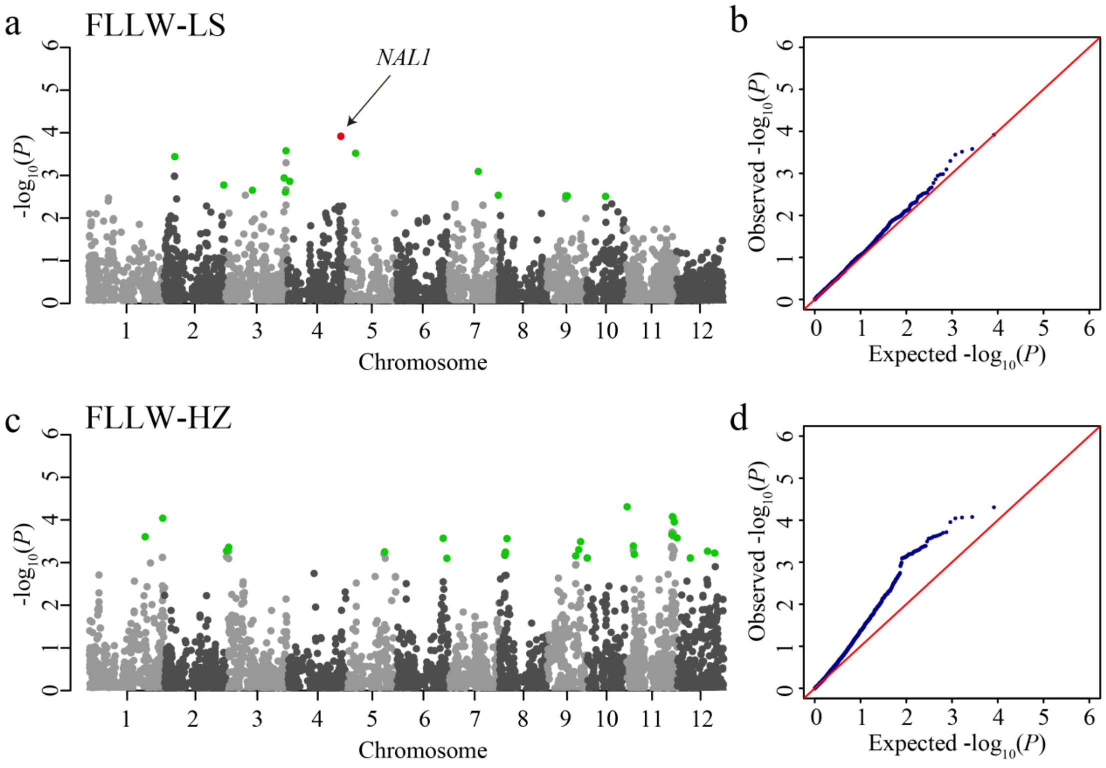

a
FLLW-LS
NAL1
-log10(P)
Chromosome
b
Observed -log10(P)
Expected -log10(P)
c
FLLW-HZ
-log10(P)
Chromosome
d
Observed -log10(P)
Expected -log10(P)

(Continued)

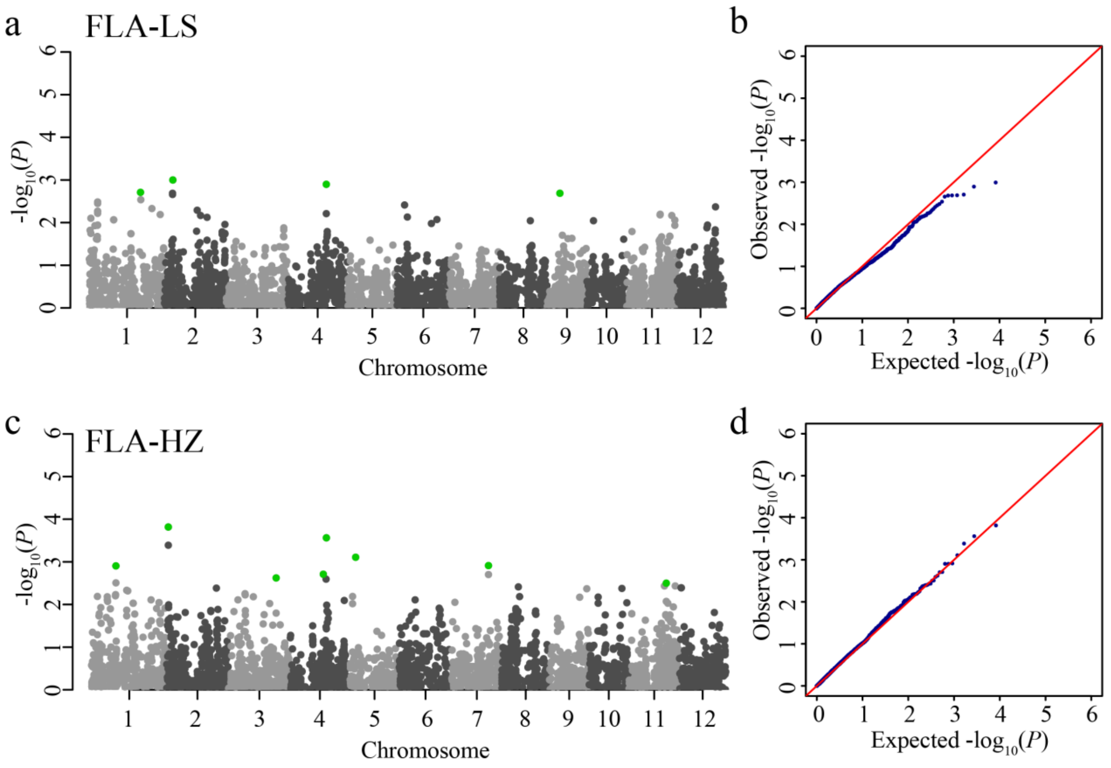

a
FLA-LS
-log10(P)
Chromosome
b
Observed -log10(P)
Expected -log10(P)
c
FLA-HZ
-log10(P)
Chromosome
d
Observed -log10(P)
Expected -log10(P)

(Continued)

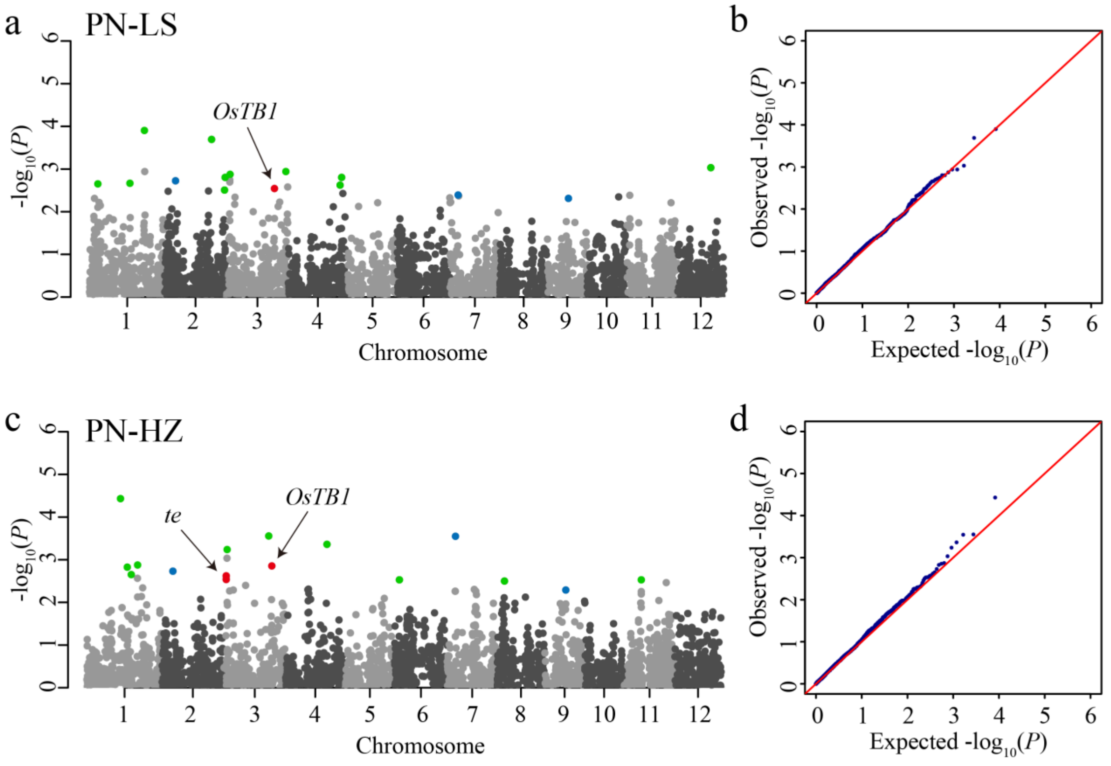
a
PN-LS
OsTB1
-log10(P)
Chromosome
b
Observed -log10(P)
Expected -log10(P)
c
PN-HZ
te
OsTB1
-log10(P)
Chromosome
d
Observed -log10(P)
Expected -log10(P)

(Continued)

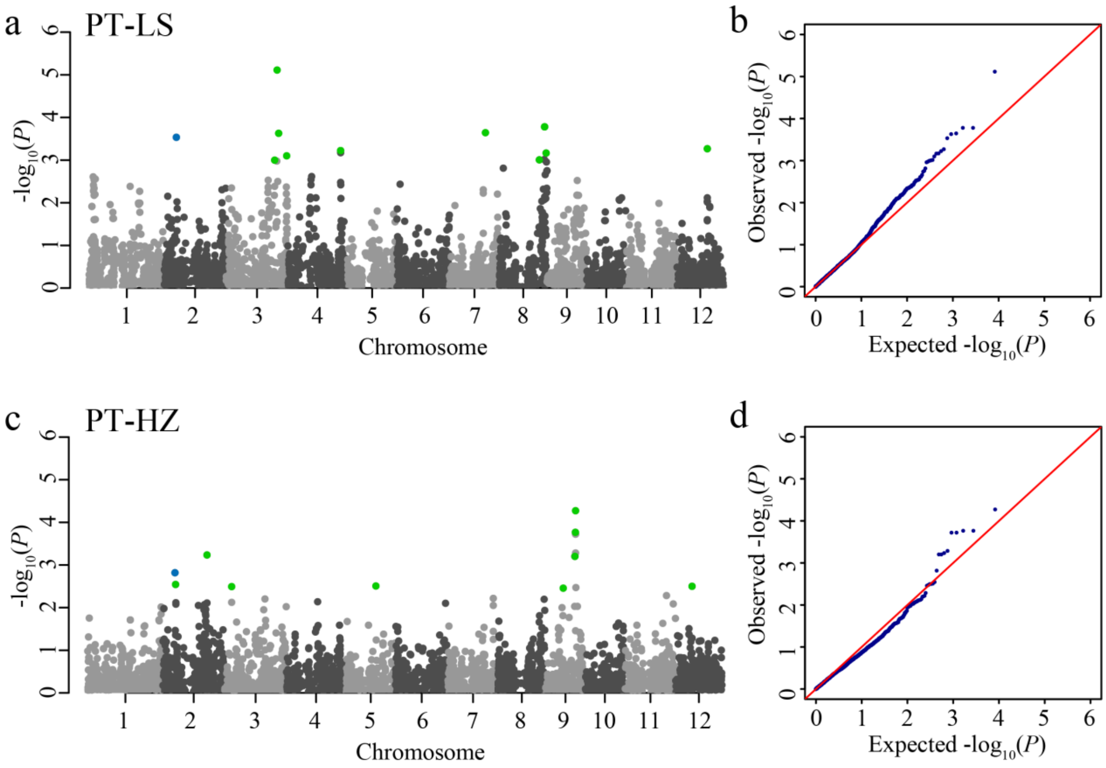
a
PT-LS
-log10(P)
0
1
2
3
4
5
6
1
2
3
4
5
6
7
8
9
10
11
12
Chromosome
b
Observed -log10(P)
Expected -log10(P)
c
PT-HZ
d

(Continued)

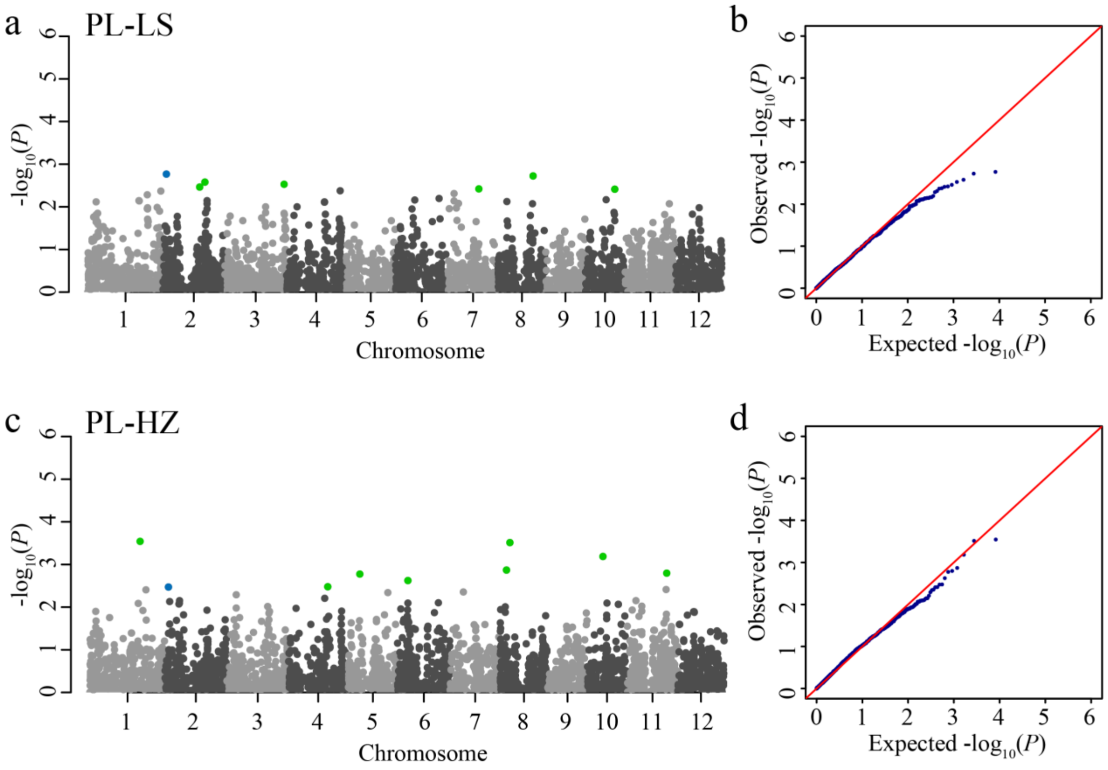

a
PL-LS
-log10(P)
0
1
2
3
4
5
6
1
2
3
4
5
6
7
8
9
10
11
12
Chromosome
b
Observed -log10(P)
Expected -log10(P)
c
PL-HZ
d

(Continued)

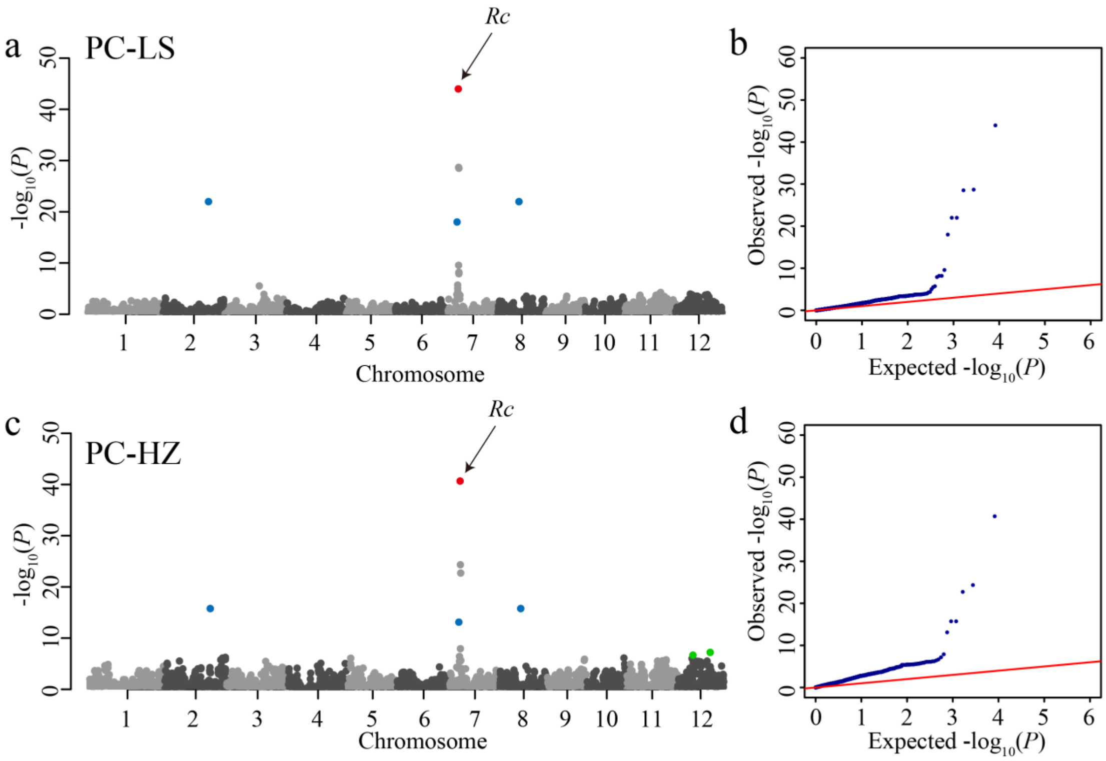

a
PC-LS
Rc
-log10(P)
0
10
20
30
40
50
1
2
3
4
5
6
7
8
9
10
11
12
Chromosome
b
Observed -log10(P)
Expected -log10(P)
0
10
20
30
40
50
60
0
1
2
3
4
5
6
c
PC-HZ
Rc
-log10(P)
Chromosome
d
Observed -log10(P)
Expected -log10(P)

(Continued)

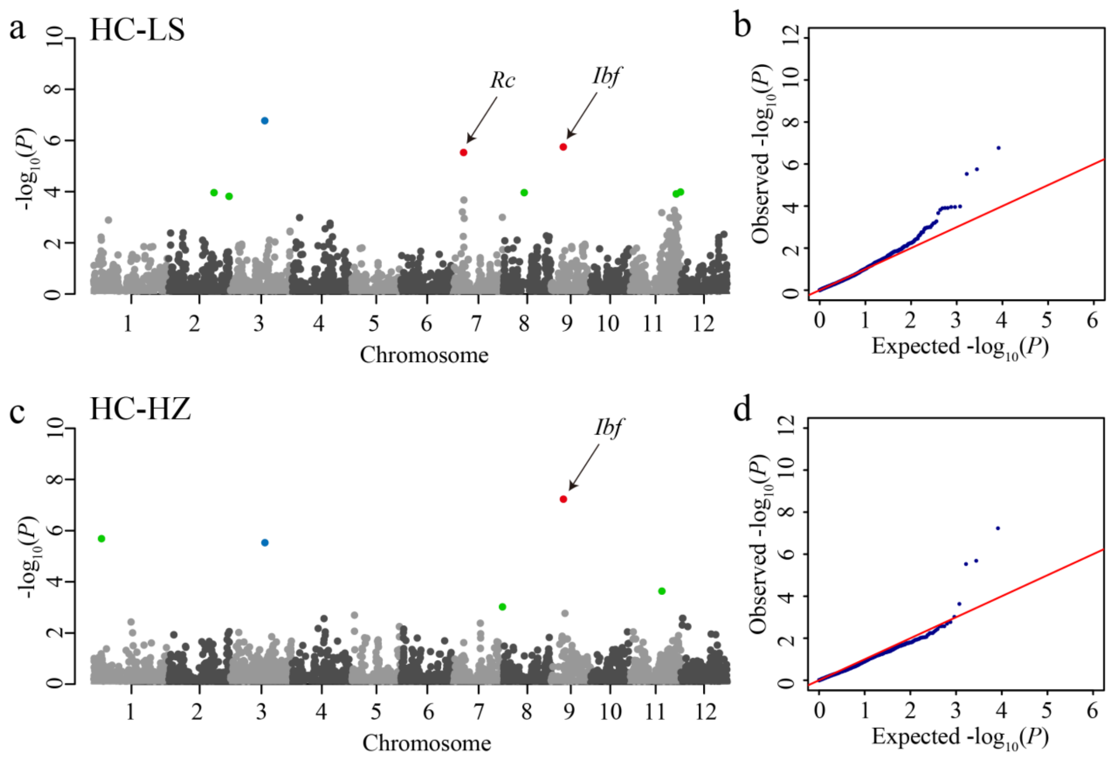

Figure S4. Manhattan and Quantile-Quantile plots of GWAS for all 12 traits in two environments. (a) and (c): Manhattan plot, showing *P*-values along the genome, calculated by mixed linear model (MLM, Q+K as covariate). (b) and (d): Q-Q plot, showing the expected null distribution of *P*-values (expected *P*-values), assuming no association, represented as a red line and distribution of *P*-values (Observed *P*-values) using MLM model represented as blue dots. Green dots indicate new significant candidate loci. Blue dots represent new significant loci detected in two environments. Red dots represent known loci. TN, Tiller number; TA, Tiller angle; PH, Plant height; FLL, Flag leaf length; FLW, Flag leaf width; FLLW, The ratio of flag leaf length and flag leaf width; FLA, Flag leaf angle; PN, Panicle number; PT, Panicle type; PL, Panicle length; PC, Pericarp color; HC, Hull color; LS, Lingshui; HZ, Hangzhou.
