# Supplementary figures and images for: Genetic variation and association mapping for 12 agronomic traits in indica rice
Source: BMC Genomics. 2015 Dec 16;16:1067. doi: 10.1186/s12864-015-2245-2 (PMC4681178; doi:10.1186/s12864-015-2245-2)

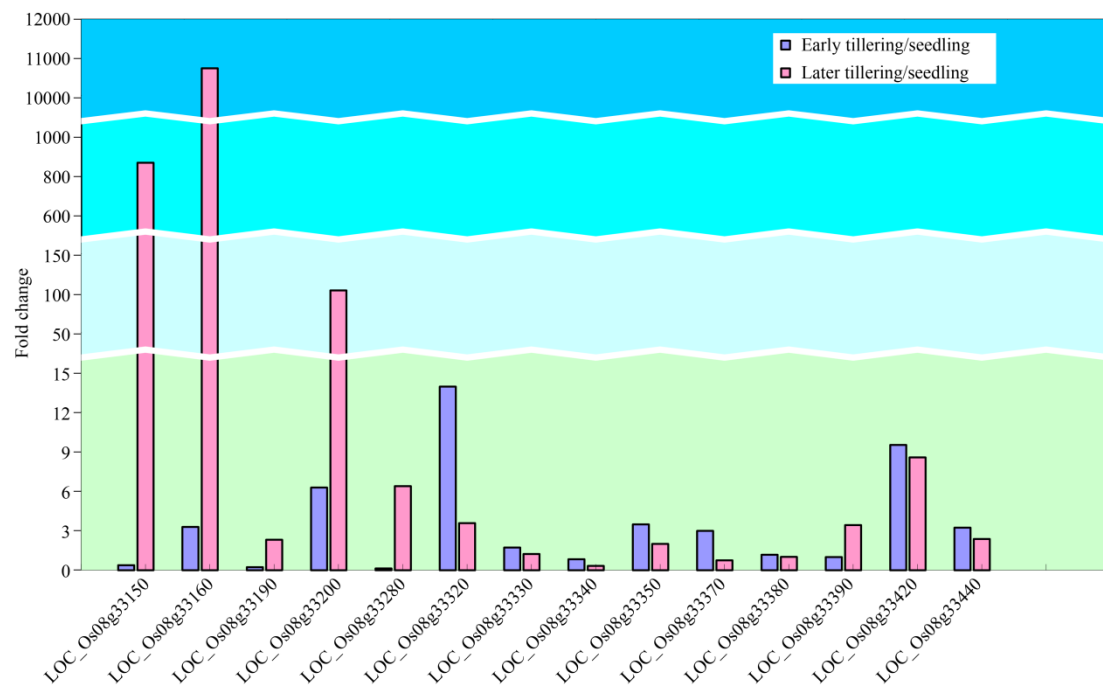

Figure S5. Fold changes of 14 tiller angle candidate genes in different tillering stages.

Supplement: Additional file 15: Figure S5. — Fold changes of 14 tiller angle candidate genes in different tillering stages. (PDF 97 kb) [file 12864_2015_2245_MOESM15_ESM.pdf]
