# Supplementary material for: Genetic variation and association mapping for 12 agronomic traits in indica rice
Source: BMC Genomics. 2015 Dec 16;16:1067. doi: 10.1186/s12864-015-2245-2 (PMC4681178; doi:10.1186/s12864-015-2245-2)
Supplement: Additional file 17: Figure S6. — Pyramid effect analysis for different numbers of elite alleles. (PDF 464 kb) [file 12864_2015_2245_MOESM17_ESM.pdf]

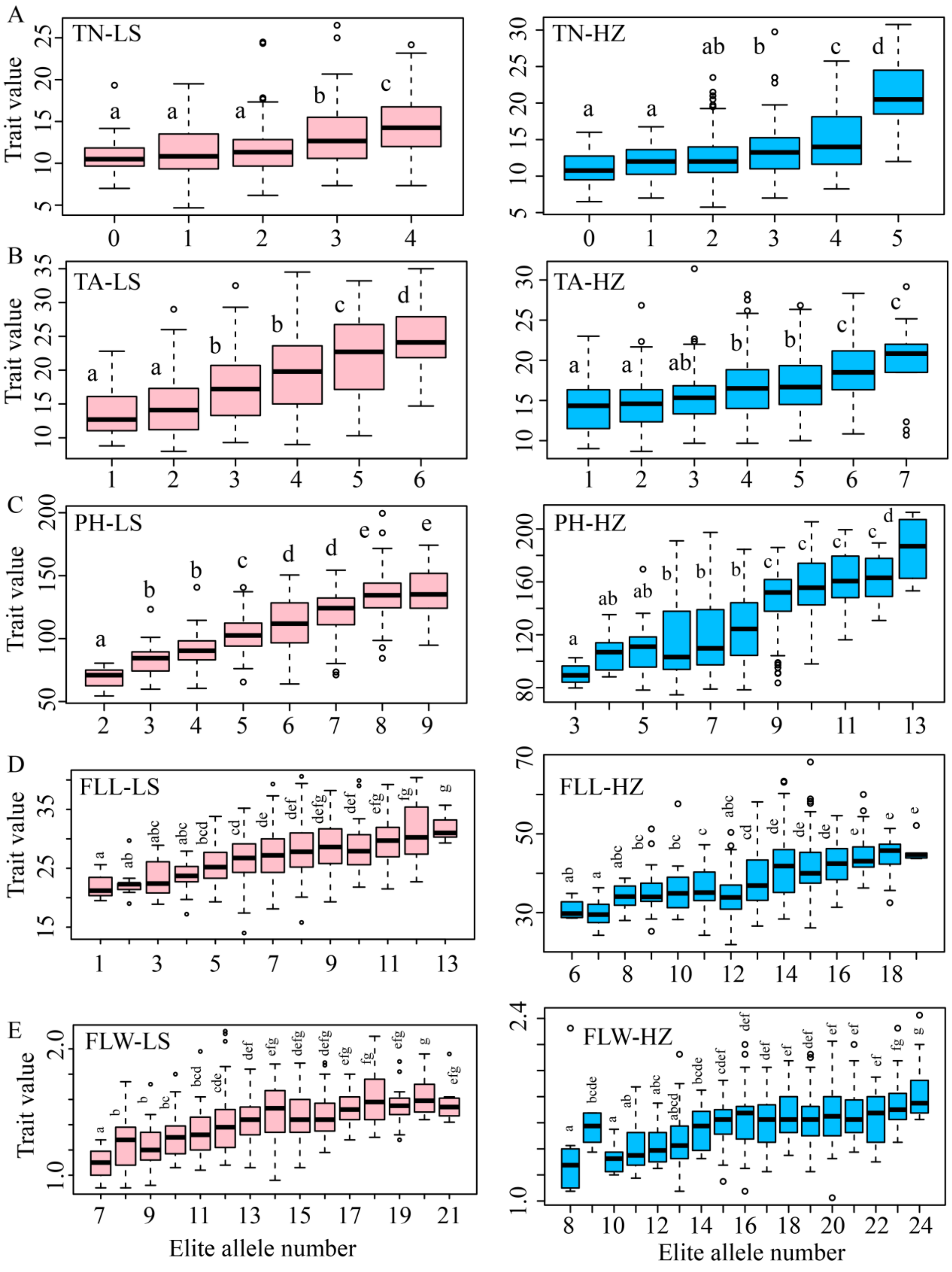
A
TN-LS
TN-HZ
B
TA-LS
TA-HZ
C
PH-LS
PH-HZ
D
FLL-LS
FLL-HZ
E
FLW-LS
FLW-HZ
Trait value
Elite allele number

(Continued)

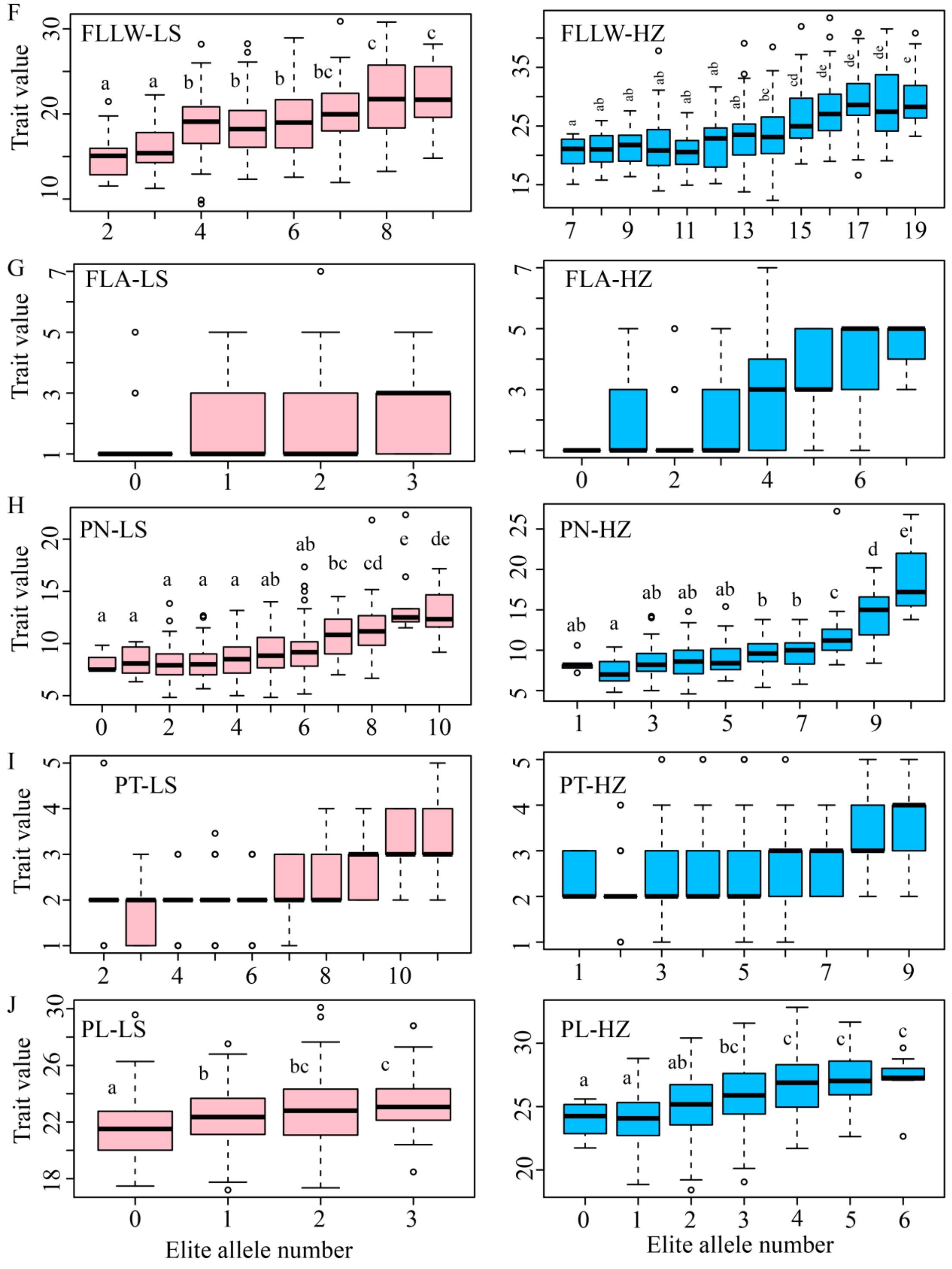

Figure S6. Pyramid effect analysis for different numbers of elite alleles. X-axis represents the number of elite alleles carried by the accessions and Y-axis represents trait mean value. Pink and blue plots represent the results in Lingshui and Hangzhou, respectively. Different lowercase letters above the plots represent Duncan multiple comparison at 0.05 level. TN, Tiller number; TA, Tiller angle; PH, Plant height; FLL, Flag leaf length; FLW, Flag leaf width; FLLW, The ratio of flag leaf length and width; FLA, Flag leaf angle; PN, Panicle number; PT, Panicle type; PL, Panicle length.
